# PIAS2-mediated blockade of IFN-β signaling: a basis for sporadic Parkinson disease dementia

**DOI:** 10.1038/s41380-021-01207-w

**Published:** 2021-07-08

**Authors:** Joana Magalhaes, Emilie Tresse, Patrick Ejlerskov, Erling Hu, Yawei Liu, Andrea Marin, Alexia Montalant, Letizia Satriano, Carsten Friis Rundsten, Eva Maria Meier Carlsen, Rasmus Rydbirk, Ali Sharifi-Zarchi, Jesper Bøje Andersen, Susana Aznar, Tomasz Brudek, Konstantin Khodosevich, Marco Prinz, Jean-François Marie Perrier, Manu Sharma, Thomas Gasser, Shohreh Issazadeh-Navikas

**Affiliations:** 1 Biotech Research and Innovation Centre, University of Copenhagen, Copenhagen, Denmark; 2 Neuronal Signaling Lab, Faculty of Health and Medical Sciences, University of Copenhagen, Copenhagen, Denmark; 3 Research Laboratory for Stereology and Neuroscience, Center for Translational Research, Bispebjerg-Frederiksberg Hospital, Copenhagen, Denmark; 4 Department of Stem Cells and Developmental Biology, Cell Science Research Center, Royan Institute for Stem Cell Biology and Technology, ACECR, Tehran, Iran; 5 Institute of Neuropathology, Signalling Research Centres BIOSS and CIBSS, Center for Basics in NeuroModulation (NeuroModulBasics), Faculty of Medicine, University of Freiburg, Freiburg, Germany; 6 Hertie Institute for Clinical Brain Research, University of Tübingen, Tübingen, Germany; 7 Centre for Genetic Epidemiology, Institute for Clinical Epidemiology and Applied Biometry, University of Tübingen, Tübingen, Germany

**Keywords:** Neuroscience, Diseases

## Abstract

Familial Parkinson disease (PD) is associated with rare genetic mutations, but the etiology in most patients with sporadic (s)PD is largely unknown, and the basis for its progression to dementia (sPDD) is poorly characterized. We have identified that loss of IFNβ or IFNAR1, the receptor for IFNα/β, causes pathological and behavioral changes resembling PDD, prompting us to hypothesize that dysregulated genes in IFNβ-IFNAR signaling pathway predispose one to sPD. By transcriptomic analysis, we found defective neuronal IFNβ-IFNAR signaling, including particularly elevated PIAS2 associated with sPDD. With meta-analysis of GWASs, we identified sequence variants in IFNβ-IFNAR-related genes in sPD patients. Furthermore, sPDD patients expressed higher levels of PIAS2 mRNA and protein in neurons. To determine its function in brain, we overexpressed PIAS2 under a neuronal promoter, alone or with human α-synuclein, in the brains of mice, which caused motor and cognitive impairments and correlated with intraneuronal phosphorylated (p)α-synuclein accumulation and dopaminergic neuron loss. Ectopic expression of neuronal PIAS2 blocked mitophagy, increased the accumulation of senescent mitochondrial and oxidative stress, as evidenced by excessive oxDJ1 and 8OHdG, by inactivating ERK1/2-P53 signaling. Conversely, PIAS2 knockdown rescued the clinicopathological manifestations of PDD in *Ifnb*^*–/–*^ mice on restoring mitochondrial homeostasis, oxidative stress, and pERK1/2-pP53 signaling. The regulation of JAK-STAT2-PIAS2 signaling was crucial for neurite outgrowth and neuronal survival and excitability and thus might prevent cognitive impairments. Our findings provide insights into the progression of sPD and dementia and have implications for new therapeutic approaches.

## Introduction

The etiology of sporadic Parkinson disease (sPD) is unknown, which complicates its treatment. However, studies of rare familial forms of this disorder have prompted its examination genomewide. All PD patients experience motor dysfunction due to loss of dopaminergic neurons in the substantia nigra (SN), correlating with pathogenic α-synuclein (α-syn) aggregates and Lewy bodies [1]. These aggregates amass in an increasingly larger area of the brain as PD progresses and causes such secondary symptoms as dementia [1, 2]. Several mutations have been identified (such as SNCA, LRRK2, DJ1, and Parkin) that are associated with familial PD [3] and some of which have been implicated in sPD [4]. However, familial forms only constitute approximately 5% of all PD cases [5, 6]—the origin of the remaining sporadic cases remains unknown.

Although sPD does not pass through familial inheritance, a genetic predisposition, in combination with environmental factors, could drive its pathology. If this predisposition is linked to several gene variants in the same pathway, the association of each gene with sPD might be weak and thus excluded in large conventional genetic association studies with strict thresholds. To include such variants, one could perform a meta-analysis of genomewide association studies (GWASs) [7] and gene set enrichment analysis (GSEA) [8].

We have reported that the lack of functional interferon-beta (*Ifnb*) or its receptor, interferon-α/β receptor (*Ifnar*), causes age-dependent development of PD-like pathology, including the accumulation of α-syn aggregates and Lewy bodies, in association with motor and cognitive dysfunction [9]. The absence of *Ifnar* from neuroectodermal cells (Nes^cre^*Ifnar*^*fl/fl*^) is sufficient to drive the pathology. *Ifnb* is a type I IFN and an immunoregulatory cytokine that participates in innate antiviral immune defense and regulates neuroinflammation [10–15]. The progression of PD is associated with neuroinflammation, including the activation of resident microglia and astrocytes, which exacerbates the pathology in the late stage of the disease [16]. However, the function of *Ifnb* signaling in neurodegeneration and neuroinflammation in sPD patients has not been determined.

The transcription and subsequent secretion of type I IFNs are induced on viral infection via Toll-like receptor signaling [11], MAVS [17], and RIG-I [18] and is orchestrated by STING-cGAS [19]. Extracellular IFN-β activates the JAK-STAT pathway on binding its receptor, IFNAR, inducing intracellular phosphorylation and nuclear translocation of STAT proteins for transcriptional initiation through interferon-stimulated responsive elements and gamma-activated sequences [20]. STATs are negatively regulated by protein inhibitors of STAT (PIASs), but their involvement in PD is unknown.

Protein inhibitor of STAT2 (PIAS2) is a negative regulator of STAT [21] and IFN-β signaling [22] and has SUMO E3 ligase activity [23]. PIAS2 was recently shown to stabilize α-syn by counteracting its ubiquitin-mediated degradation, promoting its intracellular aggregation [24].

In light of our recent findings of the relevance of defective IFNβ−IFNAR1 signaling to cause a Parkinson disease dementia (PDD)-like pathology in mice [9], we examined whether and how defective type I IFN signaling is associated with sporadic PD and its progression to dementia. Notably, genes in this pathway were significantly dysregulated in sPD patients—especially those with dementia. Moreover, we identified single-nucleotide polymorphism (SNP) variants in several of these genes that correlated with sPD, including PIAS2. PIAS2 was upregulated in the neurons of sPD patients, particularly in the brains of patients with sPDD. These findings prompted us to study how PIAS2 impacts the clinical and pathological aspects of PD—specifically, the cognitive functions that are associated with its progression to dementia. Overexpression of PIAS2 alone was sufficient to cause PD-like dementia and major pathological trademarks of PDD in mice, whereas its knockdown in vivo reversed the PD-like dementia and pathology in *Ifnb*^*–/–*^ mice, implicating it as a driver of PD pathology.

## Materials and methods

### Brain samples from sporadic PD patients and healthy controls

Human brain samples for reverse-transcription quantitative real-time (RT-q)PCR and IHC were dissected from the medial frontal gyrus, snap-frozen, and stored at −80 °C prior to analysis. Sporadic PD brain samples—sporadic Parkinson disease nondementia (sPDND) (*n* = 10), sPDD (*n* = 3), and healthy controls (HCs) (*n* = 10)—were acquired from the Harvard Brain Tissue Resource Center (Harvard Medical School, USA), Bispebjerg Hospital Brain Bank (Copenhagen, Denmark/BBH-2017-001, I-suite nr: 05190), and Netherlands Brain Bank (the Netherlands) [ethics (DNK) approval, jr. no. H-16025210]. All diagnoses were confirmed by postmortem pathology.

### Affymetrix microarray analysis

Publicly available microarray data from PDD (*n* = 13), PDND (*n* = 15), and HCs (*n* = 14) were obtained from an earlier study [PMID:18649390] [25]. The preprocessed and normalized data were acquired from the Affymetrix Human Genome U133 Plus 2.0 Array. To ensure consistency, the public data were renormalized in R using the normalize quantile function in preprocessCore. Three other independent datasets (GSE7621, GSE20141, GSE49036) on SN tissue from the postmortem brains of HCs (*n* = 25) and PD patients (*n* = 45) were pooled to validate the data from [25]. Samples were run on the Affymetrix Human Genome U133 Plus 2.0 Array and processed in R using the ReadAffy parser in affy. Preprocessing and normalization were performed with the frma package using the “robust weighted average” option for probe summarization.

Microarrays of *Ifnb*^*+/+*^ and *Ifnb*^*–/–*^ CGN cultures with or without 24-h rIFN-β treatment (100 U/ml) were set up in triplicate, and the extracted cDNA was applied to the Affymetrix Mouse Genome 430 2.0 microarray chip (SCIBLU, Affymetrix). GEO accession number GSE63815. Data were analyzed with Arraystar 3 (DNA STAR Inc.) and quantile-normalized and processed using the RMA (Affymetrix) algorithm. Data were preprocessed in R using the affy package and RMA function.

### Class prediction, gene set enrichment analysis, and interactive pathway map

BRB ArrayTools v4.5.0 (Biomedical Research Branch, NIH), using seven algorithms (1-nearest neighbor, 3-nearest neighbors, compound covariate predictor, diagonal linear discriminant analysis, nearest centroid, support vector machines, and Bayesian compound covariate classifier), was used to identify 638 differentially expressed genes (*p* < 0.01) between PDD and PDND. Leave-one-out crossvalidation, based on 1000 random permutations, was used to compute the misclassification rate. Functional annotation and pathway analysis were performed by GSEA (v2.2.2), and heatmaps were generated by extracting the core enriched genes (Molecular Signatures Database v5.1, Broad Institute). An interactive pathway map that compared gene expression in the type I IFN pathway, obtained from WikiPathways [26], in PD versus HC was created using PathVisio v3.2.1 [27]. This pathway is based on 32 publications, listed in the bibliography of the pathway. The log2-scale fold-change values were shown on a gradient from −1 to +1.

### Meta-analysis of SNPs from IPDGC

We leveraged summary statistics data from the International Parkinson Disease Genomics Consortium (IPDGC), which are described elsewhere [4]. In brief, we selected genes that were directly downstream of type I interferon receptor and performed a meta-analysis, comparing overlapping SNPs with two datasets by the IPDGC: 7,689,524 SNPs from 5333 PD cases and 12,019 HCs [4] and 7,893,274 SNPs from 13,708 PD cases and 95,282 HCs [28]. We examined the 100 kb upstream and downstream to encompass the signaling pathway and used fixed-effects meta-analysis as the primary method of analysis (Table 1 and Data File 1).

**Table 1:** Putative pathogenic rare risk variants in *PIAS2*, *JAK2*, *TYK2*, and *AKT1* associated with Parkinson disease.

*PIAS2*/chr18	Allele1	Allele2	SE	Meta_*P*
chr18:42747317	t	c	0.1619	0.04771
chr18:42719495	a	g	0.1628	0.04853

*JAK2*/chr9	Allele1	Allele2	SE	Meta *P*
chr9:4977057	t	c	0.1056	0.02126
rs7863708	t	c	0.0637	0.02209
rs7026646	a	g	0.0618	0.02856
rs7857730	t	g	0.0256	0.03138
rs2031906	t	c	0.0256	0.03521
rs10117591	a	g	0.0254	0.03582
rs7847294	a	c	0.0254	0.03743
rs10115312	t	g	0.0262	0.03766
rs7861599	t	c	0.0257	0.03947
rs4372063	a	g	0.0262	0.04055
rs2149556	t	c	0.0256	0.04097
rs7034878	a	c	0.0256	0.04097
rs9987451	t	c	0.0254	0.04242
rs10815158	a	g	0.0254	0.04322
rs3780378	t	c	0.0254	0.04322
rs10815146	a	t	0.0254	0.0441
rs10815144	a	g	0.0254	0.04417
rs7852988	a	c	0.0254	0.04417
rs7034753	a	g	0.0254	0.04491
rs4587378	t	c	0.0261	0.04607
rs1328918	a	g	0.0256	0.04644
rs4282620	a	c	0.0256	0.04671
rs7019858	t	c	0.0256	0.04671
rs7033052	c	g	0.0258	0.04939

*TYK2*/chr19	Allele1	Allele2	SE	Meta *P*
chr19:10349680	a	g	0.0713	0.007884
chr19:10349007	a	g	0.1136	0.04181

*AKT1*/chr14	Allele1	Allele2	SE	Meta *P*
rs3803304	c	g	0.0344	0.001726
chr14:104314779	a	g	0.0443	0.004011
chr14:104314770	a	g	0.0526	0.008963
rs10149779	a	g	0.0475	0.01566
rs3730358	a	g	0.0621	0.02332
chr14:104328847	a	g	0.0631	0.04163
rs2498800	t	c	0.0284	0.04245
rs2494734	c	g	0.029	0.04262
rs12590657	c	g	0.0293	0.04303
rs2498797	t	c	0.029	0.04405
rs3001371	t	c	0.0292	0.04445
rs2494733	c	g	0.0288	0.04454
rs2494731	c	g	0.0281	0.04508
chr14:104328399	a	g	0.063	0.04511
rs1130233	t	c	0.0298	0.04594

### Mice

*Ifnb*^*–/–*^ mice were backcrossed for 20 generations to B10.RIII [9] or C57BL6 mice. The *Ifnar1*^*–/–*^ and *nes*^*Cre*^*:Ifnar1*^*fl/fl*^ mice were on the C57BL6 background [13]. The wild-type (WT) animals were *Ifnb*^*+/–*^, *Ifnb*^*+/+*^, or *Ifnar*^*+/+*^ littermates. Mice were housed in standard facilities. Sex- (equal proportion male and female), age- (8–9-week old at injection), and weight-matched (28–31 g) mice were used in the experiments according to the ethical committees in Denmark and our institutional review boards (2013-15-2934-00807, 2018-15-0201-01572).

### AAV6 production

HEK293 cells (80% confluent) were transfected with pDP6 AAV6 [29] and pAAV-SYN1–mCherry-mouse PIAS2, pAAV-SYN1–mCherry-mouse mutant PIAS2_C362A_, or pAAV-SYN1-mCherry (Vector Builder) by CaCl2 method. After 72 h, cells were harvested by scraping, centrifuged at 200 g, and lysed with 5 ml Tris 0.05 M, NaCl 0.15 M, MgCl_2_ 0.005 M. Three freeze-thaw cycles were then performed. Benzonase (Invitrogen) was added to 50 U/ml and incubated for 30 min at 37 °C, and the mixture was centrifuged at 4500 g for 15 min. Supernatant was collected, and 5 ml lysis buffer was added to the cell pellet. After centrifugation at 4500 g for 15 min, the second supernatant was collected. Both supernatants were pooled and passed through 0.45- and 0.22-μm Acrodisc filters. The filtered supernatant was added to an iodixanol gradient (1.5 ml for each step from 0 to 15 to 25 to 40 and 60%) and centrifuged at 50,000 rpm for 2 h at 4 °C (70.1 Ti Rotor Beckman). The adeno-associated virus (AAV) fraction was collected between the fractions at 60 and 40%, concentrated, and buffer-exchanged to PBS by centrifugation at 2000 g at 4 °C using an Amicon 100 K filter. Finally, the AAV was passed through a 0.22 Acrodisc filter. Titration was performed by RT-qPCR for WPRE using fw 5’-CCGTTGTCAGGCAACGTG-3’ and rev 5’-AGCTGACAGGTGGTGGCAAT-3’ as primers. The titers that we obtained were 3.5e10^6^/μl for AAV mCherry, 9.0e10^6^/μl for mutPIAS2, 7.4e10^6^/μl for WT-PIAS2, and 3.0e10^6^/μl for hSNCA.

### Stereotactic injection of AAV6 PIAS2 and PIAS2 siRNA

Two-month-old WT, *Ifnar1*^*–/–*^, and *Ifnb*^*–/–*^ C57BL6 mice underwent stereotactic brain injections of AAV6 PIAS2-mCherry (pAAV{Exp}-SYN1>mCherry(ns):mPias2{NM_008602.4}:WPRE}; Vector Builder) or AAV6 PIAS2_C362A_-mCherry (pAAV{Exp}-SYN1>mCherry(ns):mPias2{NM_008602.4}*(C362A):WPRE}; Vector Builder)—with or without AAV6 human SNCA (pAAV{Exp}-SYN1>hSNCA{NM_000345.5}:WPRE}; Vector Builder). For the siRNA knockdown experiments, Accell siRNA against PIAS2 and a scrambled control were obtained from Dharmacon. AAV6 vectors were prepared as described. Isoflurane was used for general anesthesia through inhalation. Before injection 4% lidokain ointment is applied topically before a skin incision (max 0.5 cm).

Mice were placed in a stereotactic frame, and AAV or siRNA was injected using a 10-μl Hamilton syringe. Two microliters of solution was injected unilaterally or bilaterally into the SN, striatum, and prefrontal cortex; 16 days or 1 month later, their behavior was tested, and their brains were dissected. The AAV solution was infused at 2 μl/min, and the needle was left in place for an additional 45 s before being slowly retracted. Injections were performed at the following coordinates: SN: antero-posterior: −3 mm, medio-lateral: –/+1.3 mm, dorso-ventral: −3.9 mm; striatum: antero-posterior: +0.3 mm, medio-lateral: –/+2.3 mm, dorso-ventral: −2.9 mm; prefrontal cortex: antero-posterior: +1.8 mm, medio-lateral: –/+1 mm, and dorso-ventral: −2.5 mm—relative to the bregma per a stereotactic atlas.

After the injections, the mice were monitored daily and if signs of reduced well-being, such as inactivity, reduced grooming, loss of appetite and weight (more than 20%) were observed, the mice were euthanized. Typically the mice did not show any sign of inactivity or weigh loss.

### Behavioral measurements

Groups were sex and weight matched. The experimenter was blind to the genotype and conditions. All animal groups for each model were tested in a same battery. Fifteen and 30 days post injections were selected for behavioral analysis as cognitive impairments might take longer to exhibit while motor dyscoordinations are often appears earlier. Cognition assessments were privileged to be tested prior to motor tests to avoid interfering with behaviors.

An accelerating RotaRod (TSE Systems GmbH) was used to assess motor coordination as previously described [9]. After a pretraining period at 5 rpm for 1 min, the speed was increased from 5 to 40 rpm over 3 min with increment of 1 rpm every 5 s and kept at 40 rpm for another 2 min. Time before falling was recorded, with a maximum duration of 5 min for each trial. Four trials were performed with at least 10 min of rest between trials.

Cylinder test was conducted on asymmetrical forelimb use during vertical exploration to measure akinesia in mice. Climbing test was used to determine motor function by measuring their ability to stand and climb. One 5-min trial was performed for each test.

Gait test was administered to measure motor performance and loss of coordination. The fore and hind paws were painted with different dyes to distinguish footprints. Then, the mice were allowed to walk through a corridor to a safe box. The mice were trained three times on the day before the trial. On the day of the trial, various aspects of the footprints in the corridor were measured: total distance from the first to last step with each paw, stride length, and degree of overlap.

To measure cognitive impairment, the Barnes maze was applied. The Barnes maze consists of a circular platform with 20 circular holes around its circumference, under one of which lay an escape box, oriented by visual clues in front of the platform. On day 1 (day 15 post injection), the mice were subjected to one habituation trial (30 s in the center of the maze and 2 min inside the escape box) and three training trials of 180 s each (or until escape box was reached). The next day, to test spatial short-term memory, the test was administered twice (probe, 90 s) but with the box removed. Finally, at day 15 (day 30 after injection), the mice were subjected to a new round twice (probe, 90 s) to test long-term memory. An automated video tracking system, Ethovision 3.1 (Noldus Information Technology), recorded the time that elapsed to find the escape hole during the learning trials and that in the area of the escape hole during the probe tests. Zone 1 corresponds to the quarter part of the maze including goal box.

### Cell cultures

Cerebellar tissue was dissected from 7-day-old mice, and in vitro cultures were established as described [30].

Cortical neuron (CN) cultures were obtained from the cortex of 1-day-old mice, as described (Brewer and Torricelli [31]). Neurons were cultured on poly-D-lysine-coated plates in neurobasal medium (Gibco) that contained B27 (2%) and gentamicin.

SHSY5Y (Lundbeck) stock cells were kept in DMEM with GlutaMax and 10% fetal bovine serum (FBS) and plated on laminin-coated plates. Neuronal differentiation was induced with retinoic acid (1 μm) in DMEM with GlutaMax and 2% FBS for 4 days. Recombinant IFN-β (R&D Systems, UK) was added to neuronal cultures at 30 U/ml for 3 days or 100 U/ml for 24 h.

### siRNA transfection of cell cultures in vitro

siRNA-mediated knockdown was performed using the Accell SMART pool system from Dharmacon (Thermo Scientific) or Sigma, combining 3–4 siRNAs per transduction using 10 nM of each sequence. SHSY5Y cells and CNs were transfected with siRNAs in Accell or Lipofectamine 2000 (Invitrogen) per the manufacturer, incubated for 48 h, and treated with IFN-β, AZD, or control for another 24 h before analysis.

Delivery efficiency and siRNA specificity were examined by WB. The Accell nontargeting control and universal negative control 1 (UNC1; Sigma) were the siRNA controls [32].

The siRNA sequences were as follows: *STAT1*#1: 5’ CUG UGA AGU UGA GAC UGU U 3’, *STAT1*#2: 5’ CUC AUU CCG UGG ACG AGG U 3’, *STAT1*#3: 5’ CCU GAU UAA UGA UGA ACU A and *STAT1*#4: 5’ CGU AAU CUU CAG GAU AAU U 3’. *STAT2#1:* SASI_Hs01_00111824; STAT2#2: SASI_Hs01_00111825; STAT3#3: HA03445053. The siRNAs were a mixture of 10 nM of each sequence.

The target sequence of the *PIAS2* Mission® siRNA (cat. no. EHU122581) was: AGA AAA AGC CCA CCT GGA TTT GTC CTG TGT GTG ACA AAA AAG CTG CCT ATG AAA GTC TAA TAT TAG ATG GGC TTT TTA TGG AAA TTC TCA ATG ACT GTT CTG ATG TAG ATG AGA TCA AAT TCC AAG AAG ATG GTT CTT GGT GTC CAA TGA GAC CGA AGA AAG AAG CTA TGA AAG TAT CCA GCC AAC CGT GTA CAA AAA TAG AAA GTT CAA GCG TCC TCA GTA AGC CTT GTT CAG TGA CTG TAG CCA GTG AGG CAA GCA AGA AGA AAG TAG ATG TTA TTG ATC TTA CAA TAG AAA GCT CTT CTG ACG AAG AGG AAG ACC CTC CTG CCA AAA GGA AAT GCA TCT TTA TGT CAG AAA CAC AAA GCA GCC CAA CCA AAG GGG TTC TCA TGT ATC AGC CAT CTT CTG TAA GGG TGC CCA GTG TGA CTT CGG TTG ATC CTG CTG CTA TTC CGC CTT CAT TAA CAG ACT ACT CAG TAC CAT TCC ACC ATA CGC CAA TAT CA.

### Immunohistochemistry, immunofluorescence, and transmission electron microscopy

For IHC and IF, mice were perfused, and their brains were fixed in 4% paraformaldehyde (PFA) and paraffin-embedded, or their brains were dissected and snap-frozen before sectioning. In vitro neuronal cultures were fixed in 4% PFA before staining. Tissues and cells were stained as described [30].

IF images were taken with a Zeiss LSM510 confocal scanning microscope and an IN Cell Analyzer 2200 automated microscope. IHC images were taken with a NanoZoomer 2.0-HT digital slide scanner or Olympus BX51 microscope. Images were processed and quantified in ImageJ (Fiji version), IN Cell Investigator, CellProfiler, Zeiss Zen, and Adobe Photoshop.

Polyclonal and monoclonal rabbit anti-β-amyloid_1–42_ (1:50, Millipore, AB5078P; and Invitrogen, clone: H31L2, 700254, respectively), mouse anti-β-amyloid_1–16_ (1:500, clone 6E10, 803015, Biolegend, US), and biotin-labeled secondary antibodies (Vector Laboratories) were used for TEM, after which the samples were treated with 0.3% H_2_O_2_. IF staining was performed with DAPI (DAKO), rabbit anti-phosphorylated (Ser129) α-synuclein (1:300, AbCam, clone EP1646Y, Ab51252), rabbit anti-PIAS2 (1:100, Neo Scientific, A5654), mouse anti-βIII-tubulin (1:50, Santa Cruz, clone TUJ-1, sc-58888), and Congo Red solution (0.2%, Sigma-Aldrich, US) and Alexa Fluor^®^−488 and −568, streptavidin-Alexa Fluor^®^−488 and −568 (Invitrogen), biotinylated rabbit anti-sheep (Vector Laboratories), and streptavidin-CY3 (1:200, US Biological) as secondary antibodies.

### Antibodies and reagents

The primary antibodies were tyrosine hydroxylase (TH) (Millipore, WB 1:2000 IHC 1:200, abcam IF/IHC 1:25), alpha-synuclein (Cell Signaling, WB 1:3000), phospho alpha-synuclein (S129) (Abcam WB 1:1000, IF/IHC 1:100), human alpha-synuclein (Thermo Scientific IHC 1:100), PIAS2 (Neoscience, WB 1:3000, IF 1:50), rabbit anti-PIAS2 (1:100, Neo Scientific, A5654), vinculin (Sigma, WB 1:100000), ERK1/2 (Cell Signaling, WB 1:1000), phospho-ERK1/2 (Cell Signaling, WB 1:1000, IF 1:100), P53 (Abcam, WB 1:500, IF 1:100), phospho-P53 (Abcam, WB 1:500, IF 1:100), DJ1 (Abcam, WB 1:5000), OxDJ1 (Cys106) (Abcam, WB 1:10000, IF 1:100), DUPS1 (Abcam, WB 1:1000), sumo-1 (Millipore, WB 1:500), 8OHdG (Santa Cruz Technologies, IF/IHC 1:50), β-tubulin III (Abcam, IF 1:50), mouse anti-βIII-tubulin (1:50, Santa Cruz, clone TUJ-1, sc-58888), TOM20 (Santa Cruz Technologies, IF 1:50; Abcam, IF 1:100), CoxI (Abcam, IF 1:140), Optineurin (Santa Cruz, WB 1:500, IF 1:100), and Hsp60 (Santa Cruz Technologies, IF 1:50).

The secondary antibodies were biotinylated anti-rabbit (Vector Laboratories); anti-mouse, -rabbit, -goat, and -chicken coupled to Alexa Fluor-488, −568, and −633 (1:1000, Invitrogen); streptavidin-Alexa Fluor^®^−488 and −568 (Invitrogen); biotinylated rabbit anti-sheep (Vector Laboratories); and streptavidin-CY3 (1:200, US Biological). Nuclei were stained with DAPI (1:30.000, DAKO). Nissl (ThermoFisher Scientific, 1:200) was used to stain neurons.

### Quantitative real-time PCR

Total RNA was isolated using the QIAGEN RNeasy microkit (QIAGEN), reverse-transcribed, amplified, and quantified using SYBR Green (Bio-Rad). RT-qPCR reactions were run with LightCycler 480 software (Roche). Relative mRNA expression was normalized to *Gapdh*.

All murine primers were purchase from QIAGEN. The human primers are listed below. The DNA oligomers were designed and ordered from TAG Copenhagen A/S, except for the all-in-One™ RT-qPCR primers for *Ifna14*, *Ifng*, and *Tnf*, which were purchased from GeneCopoeia.

### RT-qPCR primers

▓

**Table:** 

Gene	Forward primer	Reverse primer
*STAT2*	ATTCTGCAGCATTTCCCACT	GCTCATACTAGGGACGGGAAG
*PIAS2*	TGTTGAGGTGTCAAAGCAAAA	TGATGTTCTCATCAAGCCCA
*IRF3*	TAAACGCAACCCTTCTTTGC	GATGCACAGCAGGAGGATTT
*IRF9*	CATGGCTCTCTTCCCAGAAA	AGCTCTTCAGAACCGCCTAC
*IFNB1*	TGGAGAAGCACAACAGGAGA	AACCTTTCGAAGCCTTTGCT
*IFNA14*	TTTGATTCAACTTGTGGTGGTT	TGGTTCATCATGGAAATGATTC
*IFNA17*	ATTCTTCCCATTTGTGCCAG	AATGGCCCTGTCCTTTTCTT
*IFNG*	TGTATTGCTTTGCGTTGGAC	TGACCAGAGCATCCAAAAGA
*IFNGR2*	ATCAGCGATGTCAAAGGGAG	TGACAATGCCTTGGTTTCAA
*IFNGR1*	TGGCATGATCTGGTACTCCC	CTTGTCATGCAGGGTGTGAG
*STAT1*	TGAATATTCCCCGACTGAGC	AGGAAGACCCAATCCAGATGT
*JAK1*	CTGCTCATTGTCGTTGGTTC	TGCCCTGTATGACGAGAACA
*GAPDH*	AATCCCATCACCATCTTCCA	TGGACTCCACGACGTACTCA
*TNF*	AGATGATCTGACTGCCTGGG	CTGCTGCACTTTGGAGTGAT
*TGFB1*	AAGTTGGCATGGTAGCCCTT	CCCTGGACACCAACTATTGC
*IL1B*	AAGCCCTTGCTGTAGTGGTG	GAAGCTGATGGCCCTAAACA
*PARK7*	GTGCAGTGTAGCCGTGATGT	CCTCCTGGAAGAACCACCAC
*SOD1*	GTGATTGGGATTGCGCAGTA	TGGTTTGAGGGTAGCAGATGAGT
*UCP2*	CAGCCAGCGCCCAGTACC	CAATGCGGACGGAGGCAAAGC

### Western blot

Samples were lysed in 1% Triton X-100 (Sigma) in lysis buffer [100 mM NaCl, 50 mM Tris-HCl, 1 mM EGTA, 10 mM MgCl_2_(6H_2_O)], measured for protein concentration, loaded onto 4–12% gels (NuPage^®^), and processed as described [33].

### Electrophysiology

Primary CNs from WT and *Ifnb*^*–/–*^ were cultured as described above. PIAS2 was overexpressed using AAV6 pAAV-SYN1–mCherry-mouse Pias2 or pAAV-SYN1–mCherry (Vector Builder) by CaCl2 method on day 1 of culture or knocked down by siRNA with the Accell SMART pool system (Thermo Scientific) for the last 72 h after 9–10 days in culture. SHSY5Y cells were kept in artificial cerebrospinal fluid, containing (in mM) 125 NaCl, 2.5 KCl, 26 NaHCO_3_, 2 CaCl_2_, 1 MgCl_2_, 1.25 NaH_2_PO_4_, and 25 glucose, in 95% O_2_ and 5% CO_2_ at room temperature. Glass pipettes (borosilicate, resistance 5–10 MΩ, Sutter Instruments, US) were filled with (in mM): 122 K-gluconate, 2.5 MgCl_2_, 0.3 CaCl_2_, 5.6 Mg-gluconate, 5 K-HEPES, 5 H-HEPES, 5 Na_2_ATP, 1 EGTA, 10 biocytine, Alexa Fluor^®^488 (10 µM, Sigma), and KOH to adjust the pH to 7.4.

Cells were visualized under a BW51WI microscope (Olympus, Japan). Whole-cell recordings were made in voltage clamp mode with a Multiclamp 700B amplifier (Molecular Devices, US). Recordings were sampled at 20 kHz with a 16-bit analog-to-digital converter (DIGIDATA 1440; Molecular Devices, US) and displayed with Clampex 10.2 (Molecular Devices, US). Fast transient sodium inward currents [34] were evoked by depolarizing steps of 10-mV increments, applied from −90 mV. The inward current amplitude (calculated as the difference between the peak and baseline) was maximal at 0 or +10 mV.

### Statistical analysis

Data were analyzed by unpaired two-tailed Student’s *t*-test, ANOVA (parametric) or Mann–Whitney *U* (non-parametric); correlation analysis; Shapiro–Wilk normality distribution test and Fisher’s exact test using Prism software. *P* < 0.05 was significant. Error bars are ± SEM.

## Results

### Neuronal IFNβ–IFNAR signaling is defective and associated with increased PIAS2 in patients with sporadic Parkinson disease dementia by transcriptomic analysis

**Fig. 1:**
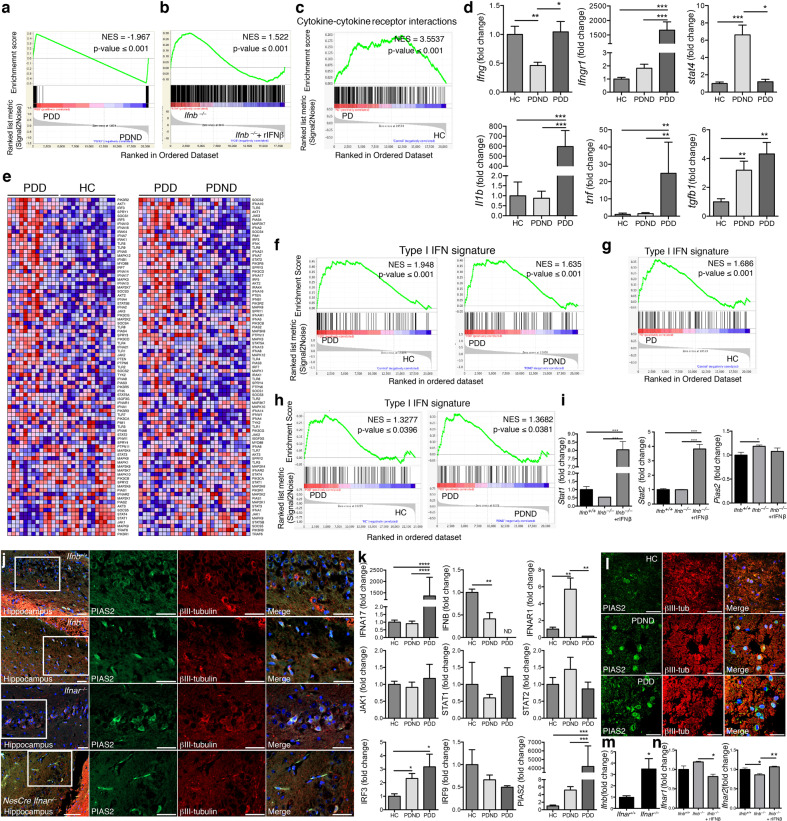
Upregulation of neuronal PIAS2, a negative regulator of *IFNβ-IFNAR signaling*, in the brains of sporadic PD patients. **a** GSEA class prediction analysis distinguishing sporadic Parkinson disease patients with dementia (sPDD; *n* = 13) from sPD patients with nondementia (sPDND; *n* = 15), used as the discovery cohort. **b** Core enriched gene signature (638 genes) of sPDD versus sPDND patients used as a class predictor of *Ifnb*^*–/–*^ neurons with and without rIFN-β treatment (100 U/ml for 24 h). **c** Enrichment plot of “cytokine–cytokine receptor interaction” pathway comparing microarrays from substantia nigra of PD patients (*n* = 45) and HCs (*n* = 25) pooled from three independent studies (GSE7621, GSE20141, and GSE49036) and used collectively as the discovery cohort. **d** RT-qPCR of medial frontal gyrus of HC, sPDND, and sPDD patients (*n* = 3–10 patients from in-house validation cohort). Graphs represent mean ± SEM and *P < 0.05; **P < 0.01; ***P < 0.001 by one-way ANOVA and Turkey’s post hoc correction test. **e** Heatmaps from GSEA of the type I IFN signature and comparing microarray data from sporadic Parkinson disease with dementia (sPDD; *n* = 13), sPD with no dementia (PDND; *n* = 15), and healthy controls (HC; *n* = 14). **f** Enrichment plots of **e**. **g**, **h** Enrichment plots of the type I IFN signature **g** comparing PDD with HC from the pooled cohorts GSE7621, GSE20141, and GSE49036. **h** Comparing PDD with PDND and HC from GSE49036. For all plots, NES: normalized enrichment score. **i** Fold-change in *Stat1, Stat2*, and *Pias2* expression from microarray comparing *Ifnb*^*–/–*^ and *Ifnb*^*+/+*^ CGNs with or without rIFN-β (GSE63815). Graphs represent mean ± SEM of *n* = 3/group; *P < 0.05 and ***P < 0.001 by one-way ANOVA. **j** IF staining for PIAS2 (green), βIII-tubulin (red); and DAPI (blue) in hippocampus of WT, *Ifnb*^*–/–*^, *Ifnar1*^*–/–*^, and *nes*^*Cre*^*:Ifnar1*^*fl/fl*^ mice. Bar, 50 μm. **k** RT-qPCR of selected genes from medial frontal gyrus regions of HC, sPDD, and sPDND. Graphs represent mean ± SEM, *n* = 3–10/group. *P < 0.05, **P < 0.01, ***P < 0.001, and ****P < 0.0001 by one-way ANOVA and Turkey’s post hoc correction test. **l** IF staining for PIAS2 (green), βIII-tubulin (red), and DAPI (blue) in the frontal cortex region of HC, sPDD, and sPDND patients. Bar, 50 μm. **m** RT-qPCR of *Ifnb* from brain of 3-month-old *Ifnar1*^*–/–*^ and *Ifnar*^*+/+*^ mice. Graphs represent mean fold-change ± SEM (*n* = 3). *P < 0.05 by Student’s *t-*test. **n** Expression of *Ifnar1* and *−2* from microarrays of *Ifnb*^*–/–*^ and *Ifnb*^*+/+*^ CGNs with or without rIFN-β (GSE63815). Graphs represent mean ± SEM of *n* = 3/group and *P < 0.05, **P < 0.01 by one-way ANOVA.

Although rare familial genes that are associated with PD have been identified, the genes that have been linked to the etiology of sPD remain poorly characterized. We tested the hypothesis that the heterogeneous group of patients with sPD and sPDD will have defects in several genes in the same signaling pathways. To this end, we analyzed data from a previously published DNA microarray study on laser-captured CNs that were obtained from sPD patients with no dementia (sPDND) (*n* = 15), sPDD patients (*n* = 13) with clinical dementia, and HCs (*n* = 14) [25]. First, we performed class prediction to determine the core enriched genes that were differentially expressed between sPDD and sPDND patients. We found 638 such genes (*P* value ≤0.01) that classified the two patient groups (Supplementary Data File 1). This signature (Fig. 1a) also allowed the distinction of *Ifnb-*deficient PD mice [9] (GSE63815) from WT controls, validating it as a relevant animal model for PD (Fig. 1b).

By GSEA of the patient dataset, the cytokine–cytokine receptor interaction pathway was the highest ranking Kyoto Encyclopedia of Genes and Genomes (KEGG) pathway that was associated with sPDD versus HCs and sPDND (Supplementary Table 1a, b). This pathway was also significantly associated with sPDND patients compared with HCs (Supplementary Table 1c), indicating that defects in cytokine signaling intensify as the disease progresses to dementia. To verify these findings, we analyzed three additional independent studies on gene expression profiles in the SN from sPD patients (*n* = 45) and HCs (*n* = 25): GSE7621 [35], GSE20141 [36], and GSE49036 [37]; the status of dementia was not indicated in the two former studies. When combining these studies, the cytokine–cytokine receptor interaction pathway was significantly regulated [*P* ≤ 0.001; normalized enrichment score (NES): 3.5537] and ranked number 1 by GSEA, underscoring its importance in PD (Fig.1c and Supplementary Table 1d).

To verify these results, we measured the mRNA levels of select genes within the cytokine–cytokine receptor interaction pathway from the cortex of a separate in-house cohort of sPD patients and HCs. *IFNG*, *IFNGR1*, and *STAT4* were highly dysregulated between HC, sPDND, and sPDD patients. *IL1B*, *TNFA*, and *TGFB1* mRNA levels were significantly higher in sPDD versus the other groups (Fig. 1d), confirming that also in our cohort, disrupted cytokine signaling correlates with the progression of PD to dementia.

Consistent with these results, by GSEA of a neuronal microarray (GSE63815) from our *Ifnb-*deficient PD mouse model [9], the cytokine–cytokine receptor interaction pathway was significantly dysregulated between *Ifnb*^*+/+*^ neurons, *Ifnb*^*–/–*^ neurons, and *Ifnb*^*+/+*^ neurons that were treated with recombinant interferon-beta (rIFN-β), as confirmed in untreated versus rIFN-β-treated *Ifnb*^*–/–*^ neurons (Supplementary Table 1e–g). Next, we applied the gene signature of *Ifnb*^*–/–*^ versus *Ifnb*^*+/+*^ neurons and *Ifnb*^*–/–*^ versus rIFN-β-treated *Ifnb*^*–/–*^ neurons in the cytokine–cytokine receptor interaction pathway to determine whether it could differentiate sPDD patients from HCs and PDND. The gene signatures from the *Ifnb*^*–/–*^ PDD mouse model distinguished sPDD patients from the other two groups (Supplementary Fig. S1a, b), demonstrating that the PD mouse model [9] mimics the genetic and pathological aspects of PD dementia. Moreover, it highlights that the absence of *Ifnb* is sufficient to disrupt other cytokines and their signaling.

All of the top signaling pathways (cytokine–cytokine receptor, JAK-STAT, RIG, and Toll-like-receptor) contain several type I interferons—IFNAs and IFNB1 (and IFNAR)—and downstream molecules, such as JAKs, STAT2, and PIAS2 (Supplementary Table 1). To determine the relevance of defective neuronal IFNβ-IFNAR signaling to PD, we generated a common type I IFN gene signature, based on the literature (Supplementary Data File 2), and compared it with patient datasets by GSEA. Heatmaps of the signature indicated that *IFNAR1*, *JAKs*, and *STAT*s were transcriptionally upregulated in sPDD patients, as were negative regulators of this pathway, in particular *PIAS2* (Fig. 1e). The type I IFN gene signature was significantly enriched in sPDD versus HCs (*P* ≤ 0.001) and sPDND patients (*P* ≤ 0.001) (Fig. 1f). When pooling the three cohorts GSE7621, GSE20141, and GSE49036, the type I IFN signature distinguished sPD from HCs by GSEA (*P* ≤ 0.001, NES: 1.686) (Fig. 1g). The GSE49036 study separated PD patients with and without dementia [37]; thus, we used it to validate the previous dataset [25], and type I IFN-related pathways appeared among the top 20 most highly regulated pathways (Supplementary Table 1h–j, marked in bold). Similarly, significant gene enrichment was observed in the sPDD versus sPDND and HC groups (Fig. 1h), verifying our previous findings.

Next, we generated an interactive signaling map of our type I IFN signature genes (PathoVisio), comparing PD patients and HCs from the pooled cohorts (GSE7621, GSE20141, and GSE49036). This analysis revealed that several genes, both activating and inhibiting genes, were differentially regulated—e.g., *IFNAR* genes were downregulated, whereas *PIAS2*, a negative regulator of *STAT2*, was upregulated in PD patients (Supplementary Fig. S1c)—thus indicating that the signaling pathway as a whole is blocked. In support of these findings, *Stat1* and *Stat2* mRNA in cultured *Ifnb*^*–/–*^ neurons were unaffected compared with WT (*Ifnb*^*+/+*^) neurons, but rIFN-β treatment induced these genes (Fig. 1i). Notably, *Pias2* mRNA was significantly upregulated in *Ifnb*^*–/–*^ neuron cultures compared with *Ifnb*^*+/+*^ ones (Fig. 1i). Moreover, neuronal cells in hippocampal and cortex tissue expressed high levels of PIAS2 in the *Ifnb*^*–/–*^, *Ifnar1*^*–/–*^, and *nes*^*Cre*^*:Ifnar1*^*fl/fl*^ (*Ifnar1*-deficient only in neuroectodermal cells) PDD models (Fig. 1j and Supplementary Fig. S1d).

Next, we verified selected genes in the type I IFN signature in our patient cohort by RT-qPCR, which confirmed their differential regulation in sPD patients and those with dementia, including *IFNA17*, *IFNB*, and *IFNAR1* (Fig. 1k). *JAK1*, *STAT1*, and *STAT2* mRNA levels were unchanged, but *IRF3* mRNA level was significantly higher and *IRF9* one was downregulated in both sPD groups (Fig. 1k). IRF9 is an important factor in IFN-β–IFNAR signaling, in which IRF9 binds to phosphorylated STAT1-STAT2 heterodimers to generate the ISG3 complex, which in turn binds to DNA and regulates gene expression [38]. *PIAS2* mRNA rose in sPDND but peaked dramatically in sPDD (Fig. 1k). Consistent with these data, βIII-tubulin^+^ CNs had more intense PIAS2 staining in both sPD groups compared with HCs (Fig. 1l). Although *IFNA17* and *IFNAR1* were higher in some sPD groups, the defective *IRF9* and increased PIAS2 in sPD patients suggested a block in type I IFN signaling. Furthermore, when *Ifnar1* or *Ifnb* was deleted in mice, neurons upregulated *Ifnb* and *Ifnar2* mRNA, respectively (Fig. 1m, n), reflecting their attempt to compensate for inadequate IFNβ-IFNAR signaling.

These findings indicate that defects in IFNβ–IFNAR-regulated signaling are linked to the development of sPD and its progression to dementia and that the heterogeneity in sPD is associated with differential expression of several genes in the type I IFN pathway. Although such genes might be differentially expressed between individuals with sPD and between early and later stages of the disease, they might cause the same outcome: blockade of IFNβ–IFNAR signaling, thus accelerating disease progression.

### Meta-analysis of sPD GWASs: sequence variants in IFNβ–IFNAR-related genes

We also performed a meta-analysis of GWAS datasets to discover sequence variants in disease-associated genes that have failed to be detected in conventional GWASs due to stringent statistical corrections for multiple comparisons [39, 40]. To determine whether gene sequence variants in IFNβ–IFNAR signaling were associated with sPD, we analyzed a GWAS from the IPDGC, comprising 5333 PD cases and 12,019 controls with genotyped and imputed data on 7,689,524 SNPs [4]. As suggested [41], we assumed that sPD risk alleles were more likely to be distributed among genes with related functions.

Thus, we studied the enrichment of functionally related genes downstream of *IFNAR*: *IFNAR1, STAT1, STAT2, JAK1, JAK2, AKT1, TYK2, SOCS1, SOCS3*, and *PIAS2*. We identified 43 nominally significant sequence variants in *PIAS2*, *JAK2, TYK2*, and *AKT1* (Table 1) that were associated with sPD patients. We also analyzed a recent PD GWAS update from the IPDGC, consisting of 7,893,274 SNPs from 13,708 PD cases and 95,282 HCs [28]. Although the accessibility analysis was more limited in this dataset, the number of PIAS2-related SNPs increased from 2 to 54 (Supplementary Data File 3), the raise could be due to an increase in genome coverage because of the availability of Haplotype Reference Consortium panel for imputation [42].

### Regulation of JAK-STAT2-PIAS2 is important for neurite outgrowth and neuronal survival and excitability

**Fig. 2:**
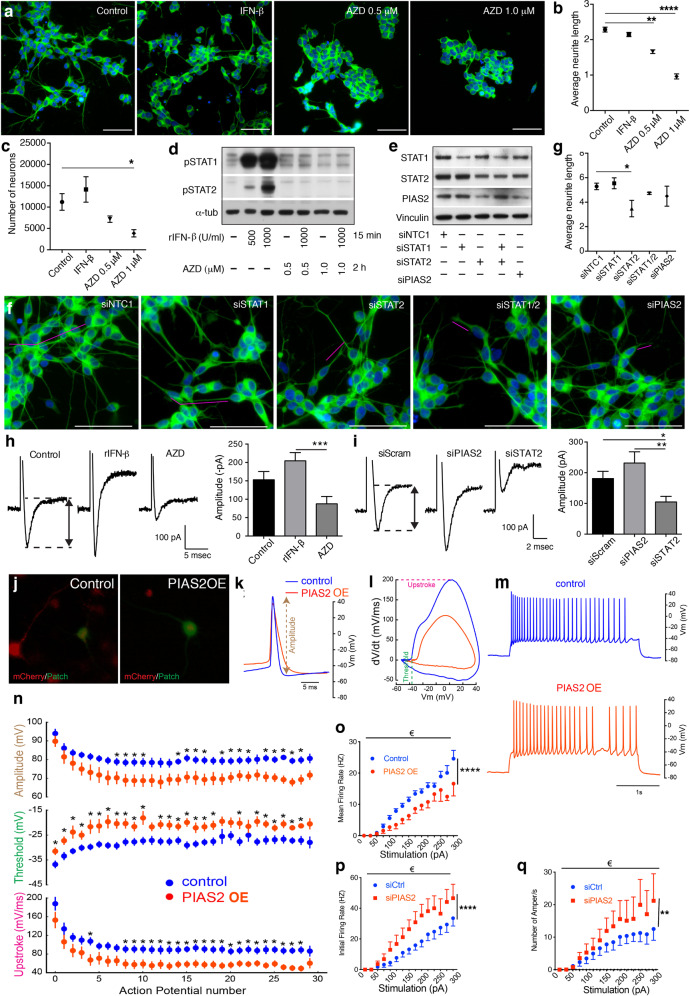
IFNβ-dependent JAK-STAT2-PIAS2 signaling controls neuronal firing. **a** Representative IF images of differentiated SHSY5Y cells treated with rIFN-β (1000 U/ml) and AZD (0.5 or 1 μM) for 24 h, showing βIII-tubulin (green) and DAPI (blue), **b** Quantified mean neurite length (estimated with βIII-tubulin), and **c** quantified neuronal survival (number of βIII-tubulin^+^DAPI^+^ cells). **d** WB of differentiated SHSY5Y cells pretreated with AZD (1 μM) for 2 h before 15-min treatment with recombinant (r)IFN-β (500–1000 U/ml). **e** WB of siRNA KD in differentiated SHSY5Y cells, NTC means nontargeting control. **f** Confocal images showing neurite outgrowth with antibodies against βIII-tubulin, and **g** quantified neurite length. Bar, 50 μm, magenta bars underline one example neurite and graphs represent mean ± SEM (*n* = 3). *P < 0.05, **P < 0.01, and ****P < 0.0001 by one-way ANOVA and Turkey’s post hoc correction test. **h**, **i** Curves represent amplitudes of the inward current evoked by a depolarizing voltage step in SHSY5Y cells after **h** treatment with rIFN-β (1000 U/ml, 24 h) or AZD (0.5 uM, 24 h) or **i** siRNA knockdown of STAT2 or PIAS2. Bar graphs represent mean ± SEM of *n* = 20 individual cells (from two independent experiments). *P < 0.05, **P < 0.01, ***P < 0.001 by Wilcoxon test. **j** Representative epifluorescence images illustrating primary cortical neurons transfected with control AAV mCherry or AAV PIAS2-mCherry (red). One mCherry-positive cell was patched in whole-cell configuration (green). **k** First action potentials generated by depolarizing current pulses in an AAV mCherry cell (blue) and AAV PIAS2-mCherry cell (orange). Note the smaller amplitude for the cell expressing AAV PIAS2-mCherry. **l** Phase plot representation of the action potentials. The PIAS2-mCherry expressing neurons had a higher threshold and a lower upstroke. **m** Examples of repetitive firing evoked in mCherry and PIAS2-mCherry expressing neurons. **n**–**q** Amplitudes, thresholds, and upstrokes measured during trains of 30 action potentials evoked by 3-s current pulses. Graphs represent mean ± SEM. *P < 0.05 by Mann–Whitney *U* test and € by two-way-ANOVA; ****P < 0.0001 both raw and column factors *n* = 10 for control and *n* = 11 for PIAS2 from three independent experiments). Note the reduced firing frequency in PIAS2-mCherry expressing neurons.

To determine the function of IFNβ–IFNAR-regulated proteins in neurons and their potential contribution to sPD pathology, we genetically and chemically modulated factors in this pathway. Using neuronally differentiated human SHSY5Y cells [43], we examined neuronal development and excitability by activating or blocking IFNAR/IFN-β signaling chemically. AZD, a chemical blocker of JAK1/2, significantly reduced both average neurite length and neuronal survival (Fig. 2a–c). Human rIFN-β treatment increased the phosphorylation of its immediate downstream target proteins, STAT1 and STAT2, which AZD blocked completely, thus validating it as a potent inhibitor of IFNAR-mediated signaling (Fig. 2d).

Next, we interfered with IFN-β/IFNAR signaling by siRNA knockdown of STAT1, STAT2, and PIAS2 (Fig. 2e). As with AZD, neurite outgrowth was negatively affected by siRNA against STAT2 (Fig. 2f, g), and both approaches significantly inhibited neuronal firing, as evidenced by the lower amplitude of the transient inward current that was evoked by a depolarizing voltage step (Fig. 2h, i). Conversely, both rIFN-β treatment and siRNA against PIAS2 significantly increased the amplitude compared with control neurons (Fig. 2h, i). In accordance, rIFN-β has been reported to influence neuronal firing in neocortical pyramidal neurons from rat brain slices in a model of multiple sclerosis [44].

To verify the impact of PIAS2 on neuronal action potential, we established primary CNs that were infected by PIAS2-mCherry or mCherry-only-expressing AAVs, and mCherry-positive neurons were selected for patch clamp; positive penetration of the patch clamp was evidenced by injected of a green fluorescent marker (Fig. 2j). PIAS2-overexpressing neurons had a lower amplitude of the initial action potential, which correlated with a higher threshold and lower upstroke (Fig. 2k, l). When examining repeated action potential, PIAS2-expressing neurons had a significantly lower amplitude, higher threshold, and lower upstroke over time, and the mean firing rate was also lower with rising stimulation pulse intensities (Fig. 2m–q).

Our data support a crucial function for PIAS2 in neurons, downstream of IFNβ–IFNAR- and STAT2-mediated signaling. Whereas IFNβ–IFNAR signaling maintains neuronal homeostasis and proper neuronal excitability, its absence or disruption, particularly by PIAS2 overexpression, interferes with neuronal action potential, neurite outgrowth, and neuronal survival.

### PIAS2 overexpression alone in the brain is sufficient to cause PD-like dementia

**Fig. 3:**
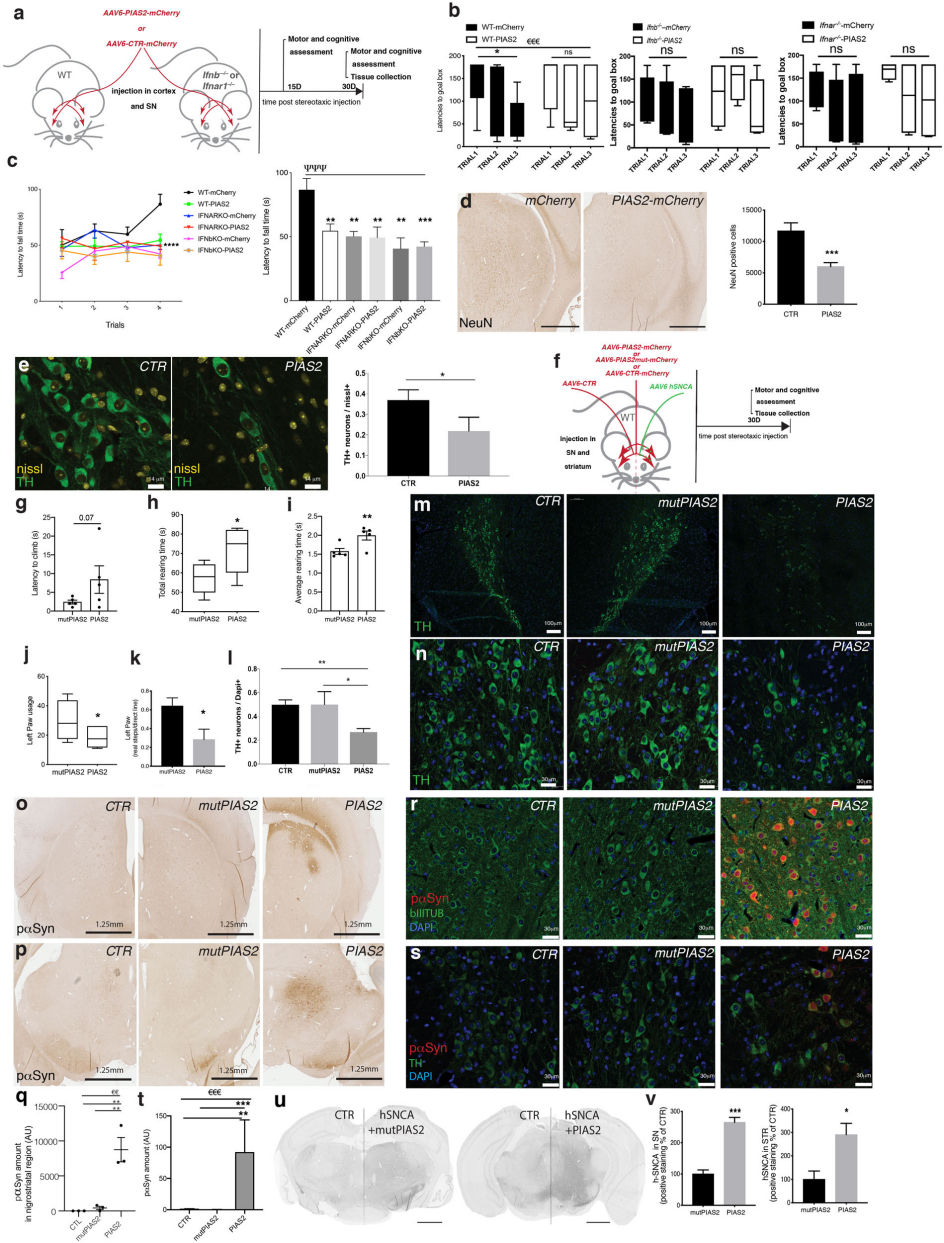
Overexpression of PIAS2 alone in neurons is sufficient to cause neurodegeneration, motor and cognitive impairments, and PD-like dementia in mice. **a** Schematic of the experimental design. WT, *Ifnb*^*–/–*^, and *Ifnar1*^*–/–*^ mice injected with AAV6 PIAS2-mCherry or AAV6 CTR (mCherry) into the substantia nigra and cortex. Tests were performed at 15 and 30 days post injection. **b** Barnes maze test. Data in seconds ± SEM. ^€€€^*P* < 0.001 by two-way ANOVA; *P < 0.05, **P < 0.01 by Student’s *t*-test. **c** Rotarod motor coordination test. Data in seconds ± SEM; ^ΨΨΨ^*P* < 0.001 by two-way ANOVA between groups and *P < 0.05, **P < 0.01, ***P < 0.001 by post hoc Dunnett’s multiple com*p*arisons test. *n* = 6 /WT-mCherry CTR, 7/WT-PIAS2-mCherry, and *n* = 4/each mCherry CTR- and PIAS2-mCherry-injected in *Ifnb*^*–/–*^ and *Ifnar1*^*–/–*^ mice; total *n* = 29. **d** NeuN IHC of the nigrostriatal region of brain of WT mice injected with AAV6 PIAS2-mCherry or AAV6 CTR mCherry and respective quantification. Data presented as number of positive cells ± SEM. ***P < 0.001 by Student’s *t*-test. *n* = 9/CTR mCherry and 12/PIAS2-mCherry. **e** IF for tyrosine hydroxylase (green) in SN in WT mouse brain injected with AAV6 PIAS2 or AAV6 CTR (mCherry) and respective quantification. Neurons stained with Nissl. Data on graph represent the number of TH^+^ neurons ± SEM, *P < 0.05 by Student’s *t*-test. *n* = 7/CTR and 6/PIAS2. **f** Schematic of the experimental design in a familial model of PD. WT mice unilaterally co-injected into the nigrostriatal region with AAV6 empty control and mCherry control on one side and human α-syn (hSNCA) together with either PIAS2-mCherry or AAV6 mutPIAS2-mCherry on the other side. **g**–**i** Motor coordination by climbing test at 16 days post injection: **g** latency to climb, **h** total rearing time, and **i** average rearing time. Data are in seconds ± SEM, *P < 0.05, **P < 0.01 by unpaired Student’s *t*-test. **j** Motor coordination by cylinder test at 30 days post AAV6 hSNCA/AAV6 PIAS2-mCherry or AAV6 hSNCA-mutPIAS2-mCherry injections showing left forepaw usage. Data in seconds ± SEM; *n* = 6/group. *P < 0.05, **P < 0.01 by unpaired Student’s *t*-test. **k** Motor performance by gait test at 30 days post AAV6 hSNCA/AAV6 PIAS2-mCherry or AAV6 hSNCA/AAV6 mutPIAS2 injections; left paw locomotion analysis by footprint. Data in cm ± SEM, *P < 0.05, **P < 0.01 by unpaired Student’s *t*-test. *n* = 6/group. **l** Quantification of **m**, **n** IF staining of TH^+^ (green) in the substantia nigra. Nuclei were stained with DAPI (blue); data in graph represent the number of TH-positive neurons ± SEM. ANOVA *P < 0.05, and **P < 0^.^01 by post hoc correction for multiple tests. *n* = 3. **o**, **p** IHC of phosphorylated (p)α-syn in brain of mice injected with AAV6 hSNCA/AAV6 PIAS2-mCherry or AAV6 hSNCA/mutPIAS2-mCherry, **o** STR, and **p** SN and **q** quantification. Scale bar is 1.25 mm. Data are mean ± SEM; *n* = 3. ^€€^*P* < 0.01 by ANOVA and **P < 0.01 by post hoc correction with Dunnett’s multiple correction test. **r**, **s** IF of pα-syn (red) and β−III-tubulin (green) in SN (**r**) and (**s**) with TH staining in SN. Nuclei were stained with DAPI; **t** quantification. Data in graph represent the number of pα-syn-positive neurons ± SEM of *n* = 3; ^€€€^*P* < 0.001 by ANOVA and **P < 0.01 and ***P < 0.001 by post hoc correction for multiple tests, applied to log-normal distribution. **u** IHC of hSNCA in SN and STR of mice injected with AAV6 hSNCA/AAV6 PIAS2-mCherry or AAV6 hSNCA/mutPIAS2-mCherry. Scale bars, 1 mm. **v** Quantification of IHC of hSNCA in SN (left) and STR (right). Bars show normalized mean percentage of control ± SEM for *n* = 4; *P < 0.05, **P < 0.01 by unpaired Student’s *t*-test.

To determine the involvement of PIAS2 in sPD, WT, *Ifnb*^*–/–*^, and *Ifnar1*^*–/–*^ mice were injected with AAVs vectors that overexpress PIAS2-mCherry or mCherry-only under the neuron-specific *SYN1* promoter in the SN and frontal cortex of the brain. Whereas WT mice that had been infected with mCherry-only AAV (CTR) performed significantly better over time in the cognitive (excluding memory) and motor learning tests—i.e., the Barnes maze and Rotarod tests, respectively—mice that overexpressed PIAS2-mCherry did not (Fig. 3a, b). As expected, mCherry-only-injected *Ifnb*^*–/–*^ and *Ifnar1*^*–/–*^ control mice did not improve with time, but PIAS2-mCherry overexpression did not aggravate the outcome further (Fig. 3c and Supplementary Fig. S 2a–d), indicating that PIAS2 and the lack of *Ifnb* or *Ifnar1* affect similar targets in this pathway. Moreover, the cognitive and motor declines due to PIAS2 overexpression in WT mice correlated with reduced TH mRNA and the loss of neurons, especially TH^+^ dopamine-producing neurons (Fig. 3d, e and Supplementary Fig. S2e).

Thus, PIAS2 overexpression in neurons is sufficient to cause motor and cognitive dysfunction, which is associated with the loss of dopaminergic neurons—the main hallmarks of PDD.

### Ectopic PIAS2 overexpression in the brain advances the disease in a familial hSCNA-PD model

To determine the effects of PIAS2 overexpression in a human α-syn (hSNCA)-induced familial model of PD, WT mice were injected unilaterally into the nigrostriatal region (SN and STR) with mCherry-only AAV (CTR) and PIAS2-mCherry or an inactive PIAS2_C362A_-mCherry mutant (mutPIAS2), which carries a mutation in the RING finger-like domain [45], together with hSNCA (Fig. 3f). Compared with mutPIAS2, PIAS2 caused significantly greater motor impairments, based on motor coordination tests, as shown in climbing with a delay to climb and an increase of rearing time and in gait test where footprint showed variations in strides, 30 days after the nigrostriatal injections (Fig. 3g–k and Supplementary Fig. S2f). This was accompanied by a severe neurodegeneration and loss of dopamine-producing (TH^+^) neurons (Fig. 3l–n). PIAS2-expressing mice experienced significant accumulation of pathogenic native phosphorylated α-syn (Fig. 3o–t) and hSNCA (Fig. 3u, v and Supplementary Fig. S2g–k) versus mutPIAS2.

These data establish that neuronal overexpression of PIAS2 in the familial hSCNA-PD model promotes the clinical and pathological manifestations of PD; accumulation of pathological forms of phosphorylated α-syn and reduction in the number of dopaminergic neurons.

### Ectopic PIAS2 overexpression in neurons blocks mitophagy, triggers mitochondrial accumulation and oxidative stress, causing oxDJ1 to inhibit ERK1/2-P53 activity

**Fig. 4:**
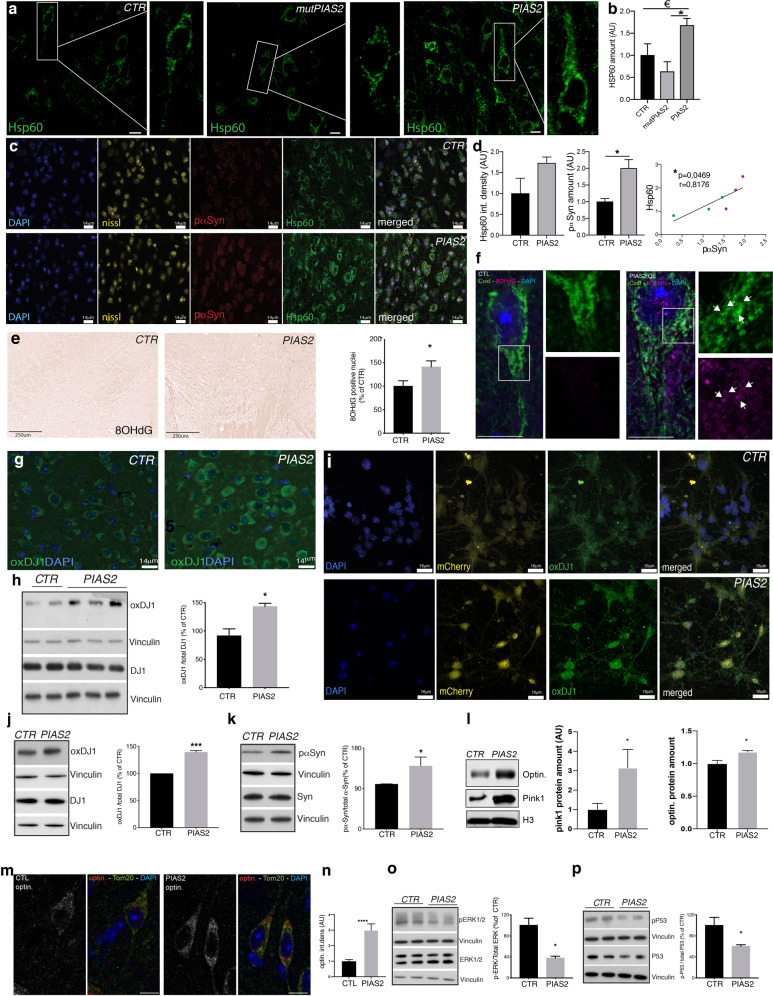
Ectopic expression of PIAS2 leads to dysregulated pP53, mitophagy block, mitochondrial accumulation, and oxidative stress. **a** IF of Hsp60 (green) and DAPI in AAV6 hSNCA/AAV6 PIAS2-mCherry- or AAV6 hSNCA/mutPIAS2-mCherry or CTR (mCherry) injected unilaterally into mouse brains. Scale bar is 14 um. **b** Quantification of Hsp60. Bars show mean ± SEM of *n* = 3; ^€^*P* < 0.05 by ANOVA and **P < 0.01 by post hoc correction for multiple tests. **c**, **d** IF of Hsp60 (green), phosphorylated (p)α-Syn (red), nissl (yellow), and DAPI (blue) in brain of mice injected with AAV6 PIAS2-mCherry or AAV6 mCherry control. Scale bar is 14 um (**c**). **d** Quantification of Hsp60 (left) and pα-syn (right) and correlation between Hsp60 and pα-syn (below). Bars show mean ± SEM; *P < 0.05 by unpaired Student’s *t*-test. *n* = 3/group. Pearson’s correlation/simple linear regression test, *n* = 9/group; mCherry controls are in turquoise and PIAS2-mCherry is in purple. **e** IHC and quantification of 8OHdG in brains of WT mice overexpressing PIAS2-mCherry or CTR mCherry. Data represent mean number of positive nuclei and are presented as % of control. Scale bar, 250 μm. *P < 0.05 by unpaired Student’s *t*-test; *n* = 6/group. **f** IF for Tom20 and 8OHdG in striatum of WT mice overexpressing PIAS2-mCherry or CTR mCherry. Arrows indicate colocalization regions of 8OHdG and CoxI. Scale bars equal 10 microns. **g** IF for oxidized DJ1 in WT mice brain overexpressing mCherry or PIAS2-mCherry. Nuclei stained with DAPI. Scale bar 14 um. **h** WB of oxDJ1 and total DJ1 for CTR and PIAS2 expression. Vinculin was used as a loading control. Data represent % of control ± SEM of *n* = 3; ***P < 0.001 by Student’s *t*-test. **i** Expression of AAV6 PIAS2-mCherry or CTR (mCherry/yellow) in primary cortical neurons (CN) and IF staining for oxidized DJ1 (green) and nuclei stained with DAPI (blue). Scale bar, 16 μm. **j, k** WB of **j** oxidized DJ1 and total DJ1 and **k** pα-syn and total α-syn .**k ** in CNs overexpressing PIAS2-mCherry or mCherry CTR. Vinculin was used as a loading control. Data represent % of control ± SEM; *P < 0.05 by Student’s *t*-test. *n* = 3. **l** WB for optin. and PINK1. H3 is used as a loading control. Data represent % of control ± SEM, *P < 0.05 by Student’s *t*-test. *n* = 3. **m **Staining for optin. and Tom20. **n **Quantification of l. WB of, **o** pERK1/2 and total ERK1/2, and **p** pP53 and total P53 in WT mouse brains overexpressing PIAS2-mCherry or mCherry CTR. Vinculin is used as a loading control. Data represent % of control ± SEM, *P < 0.05 by Student’s *t*-test. *n* = 3–4.

Next, we examined the molecular pathways by which PIAS2 affects neuronal function in vivo and in vitro. PD pathology is associated with mitochondrial dysfunction and oxidative stress, prompting us to assess these parameters further [46, 47]. Mitochondria accumulated significantly in neurons when PIAS2-mCherry was overexpressed (OE) in vivo in the brain alone or with hSCNA; this was associated with the build-up of phosphorylated α-syn (Fig. 4a–d and Supplementary Fig. S2i, j).

Rare mutations in DJ1 (PARK7) and loss of its function cause familial PD [48]. DJ1 regulates transcription and is an antioxidative stress sensor of mitochondria [49]; furthermore, it is a target of PIAS2 [50]. Consistent with these findings, PIAS2-mCherryOE in WT mice was sufficient to induce oxidative stress, as evidenced by the elevated levels of 8OHdG (Fig. 4e), affecting mtDNA (mitochondrial DNA) oxidization as 8OHdG colocalizes with mitochondrial protein CoxI (Fig. 4f). In support, PIAS2-mCherryOE in the brain also increased levels of oxidative (ox)DJ1 (Fig. 4g, h) and mRNA for *ucp2* and *park7* (the gene name for DJ1) but not *sod1* (Supplementary Fig. S2l). These findings were verified in primary CN cultures. PIAS2 expression in CNs, reflected by increased levels of PIAS2-mCherry, effected mitochondrial accumulation, upregulated oxDJ1 and phosphorylated α-syn (Fig. 4i–k).

We have reported previously that lack of neuronal type I IFN signaling is causing autophagy block, and thereby resulting in intracellular pα-syn accumulation [9], and here we observed mitochondrial accumulation in brain and CN (Fig. 4a–d and Supplementary Fig. S2m, n) and increased oxDJ1 as a consequence of PIAS2OE (Fig. 4g–k). We therefore investigated if PIAS2OE was dysregulating mitophagy, a selective autophagy process that removes damaged mitochondria. Consistent with mitophagy block, we observed that autophagy/mitophagy marker LC3II and LC3II/I ratio were increased (Supplementary Fig. S2o, p) and mitophagy specific marker Pink1 and mitophagy adaptor optineurin (optin) were significantly elevated in the brain of PIAS2OE mice (Fig. 4l–n).

Oxidative stress is reported to induce ERK-P53 signaling pathway [51] that is also shown to play a distinct role in regulating cell-cycle and death [52]. Moreover, ERK and nuclear P53 activation are reported to induce cellular autophagy [53]. We indeed identified P53 pathway as a result of active IFNβ-induced signaling in neurons (Supplementary Table 1f), and it was also among the top 6 significantly dysregulated KEGG pathways between PDD and HC (Supplementary Table 1h), as well as the top 20 in PDND vs. HC (Supplementary Table 1i). Finally, we investigate how PIAS2-mCherry overexpression in mouse brain might impact activation of ERK1/2 and P53 signaling pathway. We found that the rise in oxDJ1 (Fig. 4g–k) was negatively associated with activated/phosphorylated (p)ERK1/2 and pP53 in the brain of PIAS2OE mice (Fig. 4o, p). Of note, neurotoxicity and mitochondrial dysfunctions were not induced by mutPIAS2 control, despite a moderate but not significant impact of PIAS2 on sumoylation, SUMO1 levels (Supplementary Fig. S2q).

In conclusion, overexpression of PIAS2 in neurons causes pathology by triggering mitophagy block resulting in damaged mitochondrial accumulation and elevated oxidative stress, followed by increased oxidation of DJ1 and in turn inhibiting ERK1/2 and P53 by reducing their phosphorylation.

### PIAS2 knockdown rescues Parkinson disease-like dementia in *Ifnb*^–/–^ mice

**Fig. 5:**
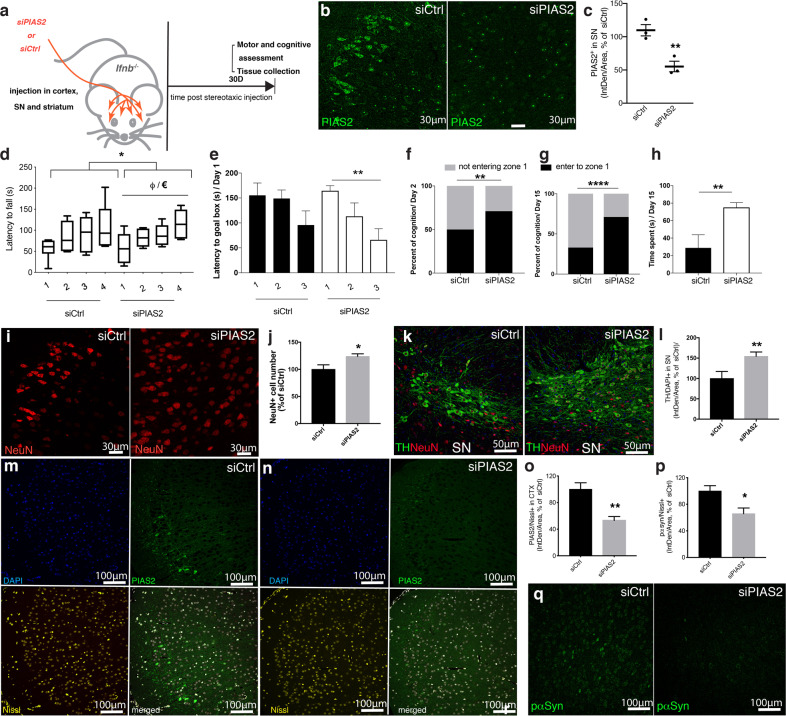
Brain-specific knockdown of *PIAS2* reverses the neurodegenerative process and prevents motor and cognitive impairments in *Ifnb*^*–/–*^ mice. **a** Schematic of experiment showing injection sites of siRNA knockdown of *PIAS2* (siPIAS2) or control (siCtrl) by in vivo stereotactic injection in the frontal cortex and nigrostriatal regions in *Ifnb*^*–/–*^ brains and behavioral testing days. **b**, **c** Assessment of knockdown efficiency by IF. **b** IF of PIAS2 (green) in the SN and **c** quantification in Nissl^+^ neurons in the SN. **P < 0.01 by two-tailed unpaired Student’s *t*-test. *n* = 3/group. **d** Rotarod motor coordination test ± SEM; *P < 0.05 by two-way ANOVA comparing siCtrl- and siPIAS2-treated groups (*n* = 6–7) and ^€^*P* < 0.05 by repeated measure one-way ANOVA in *siPIAS2*-treated *Ifnb*^*–/–*^ mice. One-way ANOVA showing ^φ^*P* < 0.05 by paired *t*-test between the 1st and 4th trials in *siPIAS2*-treated *Ifnb*^*–/–*^ mice. **e**–**h** Barnes maze test. **e** Data latency to goal box in seconds ± SEM. **P < 0.01 by two-tailed paired *t*-test in siPIAS2-treated group indicating improvement from the 1st to 3rd trial (*n* = 6–7). **f**, **g** Data are percentage of cognition (entering zone 1 or not) during probe days 2 and 15 post training day 1. **P < 0.01 and ****P < 0.0001 by two-tailed unpaired *t*-test between siPIAS2- and siCtrl-treated *Ifnb*^*–/–*^ groups (*n* = 6–7). **h** Cumulative time spent in zone 1. Data ± SEM; *P < 0.05 by two-tailed Mann–Whitney test between siPIAS2- and siCtrl-treated *Ifnb*^*–/–*^ groups (*n* = 6–7). **i**–**o** IF of PIAS2 (green) alone or co-stained with Nissl, NeuN, and DAPI (blue) and TH co-stained with NeuN in siPIAS2- vs. siCtrl-treated groups. **i** IF of NeuN and **j** quantification of NeuN^+^ neurons in the CTX. Graph represents intensity of positive staining/area of siPIAS2 normalized to siCtrl. *P < 0.05 using two-tailed unpaired Student’s *t*-test. *n* = 10/group. **k** IF of TH (green), NeuN (red), and DAPI staining in SN. **l** IF quantification of TH^+^DAPI^+^ neurons. Graph represents intensity of double-positive cells in siPIAS2 normalized to siCtrl^.^ **P < 0.01 by unpaired Student’s *t*-test; *n* = 8–9/group. **m**, **n** IF of PIAS2 (green) and Nissl (yellow) in CTX of siPIAS2 brains normalized to siCtrl group. **o** Quantification of PIAS2 in CTX. **P < 0.01 by two-tailed unpaired Student’s *t*-test. *n* = 4/group. **p**, **q** IF of pα-syn (green) in SN and quantification in Nissl+ neurons. *P < 0.05 by two-tailed unpaired Student’s *t*-test. *n* = 3/group.

To determine whether PIAS2 is a major driver of the clinicopathological manifestation of PDD in *Ifnb*^*–/–*^ mice, we injected them with AAV that expressed siRNA against *PIAS2* under the neuron-specific *SYN1* promoter, into the nigrostriatal region and frontal cortex, and performed behavioral tests 16–30 days later (Fig. 5a). This resulted in about 50% reduction of PIAS2 expression in the injected regions (Fig. 5b, c) and a moderate although not significant SUMO1 reduction (Supplementary Fig. S3a). In contrast to control siRNA (siCtrl)-injected *Ifnb*^*–/–*^ mice, *Pias2* knockdown animals improved significantly in motor abilities (Fig. 5d) as shown by rotarod tests, and in cognition as shown by Barnes maze test over three trials (Fig. 5e). Consistent with this finding, they spent more time in zone 1, where the escape exit was placed on probe Days 2 (Fig. 5f) and 15 (Fig. 5g, h), indicating cognition improvements in short-term and long-term spatial memory. In vivo knockdown of *Pias2* preserved the number of NeuN-positive (Fig. 5i, j) and TH-positive dopamine-producing neurons (Fig. 5k, l) in the SN. Comparable results were obtained in the cortex where the significant reduction in PIAS2 was associated with decline in the intracellular pα-syn (Fig. 5m–q).

These data verify that high neuronal PIAS2 levels correlate with cognitive impairments, similar to their association with dementia in PDD patients, and that eliminating neuronal PIAS2 reverses these deficiencies and neurodegenerative processes.

### PIAS2 knockdown reverses PDD pathology by preventing mitochondrial oxidative stress and restoring pERK1/2-pP53 signaling

**Fig. 6:**
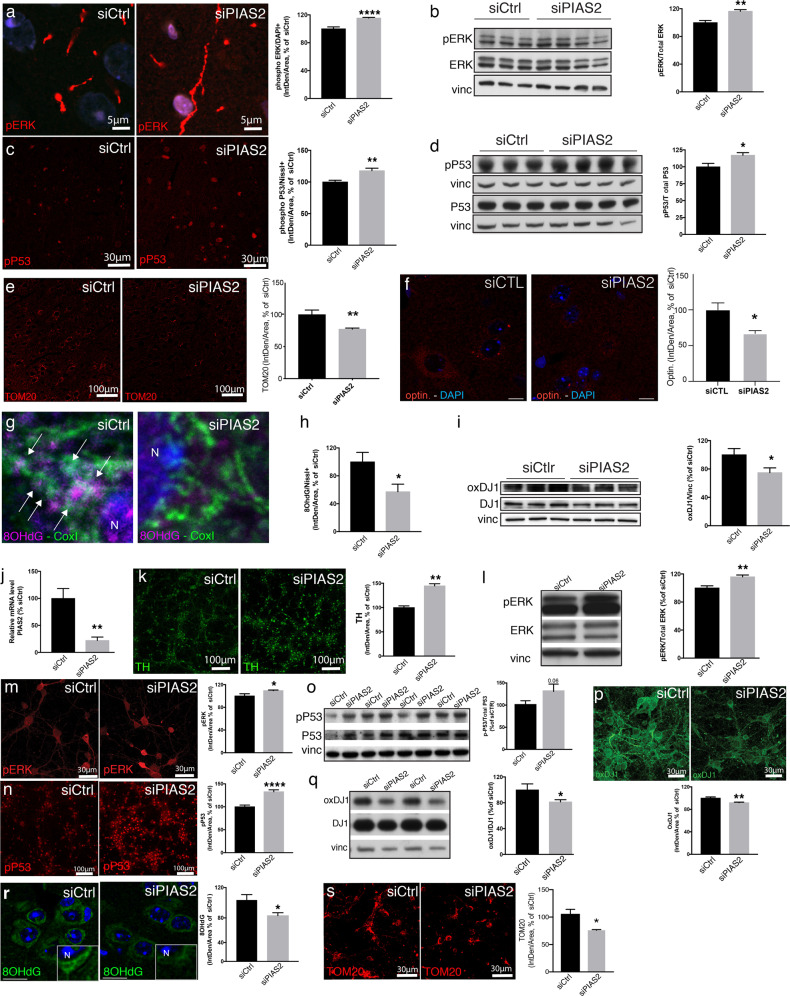
siPIAS2 reverses *Ifnb*^*–/–*^ neuronal defects by reducing oxidation of DJ1 and improving neuronal pERK- and pP53-mediated mitochondrial and neuronal homeostasis. **a**–**i** siRNA knockdown of PIAS2 (siPIAS2) or control (siCtrl) by in vivo stereotactic injection in the frontal cortex and nigrostriatal regions in *Ifnb*^*–/–*^ brains. **a** IF staining for phosphorylated (p)ERK1/2 (red) and DAPI (blue) in prefrontal cortex of *Ifnb*^*–/–*^ PDD mouse model brain after knockdown of PIAS2 or control. Graph represents mean fluorescence intensity in DAPI-positive cells, normalized to scrambled control; ****P < 0.0001 by Student’s *t*-test. *n* = 9/group. **b** WB for pERK1/2 and total ERK1/2 in *Ifnb*^*–/–*^ mouse brains after knockdown of PIAS2 or control. Vinculin was used as a loading control and values were normalized to scrambled control ± SEM; **P < 0.01 by Student’s *t*-test. *n* = 3–4/group. **c** IF staining for pP53 in prefrontal cortex of *Ifnb*^*–/–*^ mouse brain after knockdown of PIAS2 or scrambled control. Graphs represent mean fluorescence in pP53^+^Nissl^+^ neurons normalized to scrambled control; **P < 0.01 by Student’s *t*-test. *n* = 7–9/group. **d** WB for pP53 and total P53 in *Ifnb*^*–/–*^ mouse brain after knockdown of PIAS2 or control. Vinculin was used as a loading control and values were normalized to scrambled control ± SEM; *P < 0.05 by Student’s *t*-test. *n* = 3–4/group. **e** IF staining for TOM20 (red) and quantification of int.dens. in SN. **P < 0.01 by two-tailed unpaired Student’s *t*-test. *n* = 5/group. **f** IF staining for Optin. (red) in striatum and quantification. Scale bars equal 10 microns. Graph represents intensity of 30 neurons in siPIAS2 normalized to siCtrl. *P < 0.01 by two-tailed unpaired Student’s *t*-test. *n* = 3/group. **g** IF staining of CoxI and 8OHdG. White arrows indicate colocalization region. Scale bars equal 1 micron. **h** Quantification of IF staining for 8OHdG in striatum and prefrontal cortex of *Ifnb*^*–/–*^ mouse brain after knockdown of PIAS2 or scrambled control. Graph represents mean fluorescence intensity of 8OHdG in Nissl^+^ neurons, normalized to scrambled control; *P < 0.05 by Student’s *t*-test. *n* = 7/group. Images are shown in Supplementary Fig. S3. **i** WB for oxidized DJ1 and total DJ1 in *Ifnb*^*–/–*^ mouse brain after knockdown of PIAS2 or scrambled control. Vinculin was used as a loading control and normalized to scrambled contro l± SEM; *P < 0.05 by Student’s *t*-test. *n* = 6/group. **j**–**s** siRNA knockdown of PIAS2 (siPIAS2) or control (siCtrl) primary CN **j** RT-qPCR showing fold-change in *PIAS2*. Data are normalized to scrambled control ± SEM; **P < 0.01 by Student’s *t*-test. *n* = 3/group. **j** IF staining of TH. Graph represents mean fluorescence intensity and are normalized to scrambled control ± SEM; **P < 0.01 by Student’s *t*-test. *n* = 3/group. **k** Representative WB for pERK1/2 and total ERK1/2. Vinculin was used as a loading control, and data are normalized to scrambled control ± SEM; **P < 0.01 by Student’s *t*-test. *n* = 3–4/group. **l** IF staining for pERK1/2. Graph represents mean fluorescence intensity in pERK1/2^+^DAPI^+^ cells, normalized to scrambled control; *P < 0.05 by Student’s *t*-test. *n* = 3–5/group. **m** IF staining for pP53. Graphs represent mean fluorescence in pP53^+^ cells, presented as % of control; ****P < 0.0001 by Student’s *t*-test. *n* = 3/group. **n** WB for pP53 and total P53. Vinculin was used as a loading control, and data are normalized to scrambled control ± SEM. *P* = 0.06 by Student’s *t*-test. *n* = 4/group. **o** Representative WB for phosphorylated and pan P53. Data are normalized to scrambled control ±SEM. *P < 0.05 by Student’s *t*-test. **p** IF staining for oxidized DJ1. Graph represents mean fluorescence intensity of oxDJ1 in Nissl^+^ neurons, presented as % of control; **P < 0.01 by Student’s *t*-test. **q** Representative WB for oxidized and total DJ1. Data are normalized to scrambled control ±SEM. *P < 0.05 by Student’s t-test. **r** IF staining for 8OHdG. Graph represents mean fluorescence of 8OHdG in Nissl^+^ neurons, normalized to scrambled control. Close up shows non-nuclear localized 8OHdG positive staining. N indicates nuclei. *P < 0.05 by Student’s *t*-test. *n* = 7/group. **s** IF staining for TOM20. Graph represents mean fluorescence intensity normalized to scrambled control; **P < 0.01 by Student’s *t*-test. *n* = 5/group.

Next, we examined whether siRNA-mediated knockdown of *Pias2* in the brain of *Ifnb*^*–/–*^ mice mitigates the signals that contribute to mitochondrial pathology. siPIAS2 enhanced ERK1/2 activation—pERK1/2 increased and translocated primarily to the nucleus, and the pERK/ERK ratio rose significantly (Fig. 6a, b). Similarly, knockdown of PIAS2 upregulated pP53 in neurons (Fig. 6c, d), indicating the reversal of the defects that are observed in *Ifnb*^*–/–*^ neurons.

The pathology of PD is linked to greater oxidative stress [54], contributing to the progressive loss of neurons. Accordingly, in our PDD model, the control *Ifnb*^*–/–*^ group accumulated damaged mitochondria, with high levels of 8OHdG, in particular affecting mtDNA, and DJ1 oxidation (Fig. 6e–i and Supplementary Fig. S3b, c). The *Pias2* knockdown lowered the mitochondrial mass (Fig. 6e and Supplementary Fig. S3b, c), the amount of optin (Fig. 6f), and the oxidative marker 8OHdG colocalized with CoxI (Fig. 6g, h and Supplementary Fig. S3b), and the ratio between DJ1 and oxidized DJ1 (Fig. 6i and Supplementary Fig. S3c). Because it has been reported that CNs in culture express TH [55], these results were verified in primary *Ifnb*^*–/–*^ CN cultures, in which knockdown of PIAS2 upregulated TH in *Ifnb*^*–/–*^ neurons (Fig. 6k) and significantly increased pERK1/2 and pP53 levels (Fig. 6l–o). These events in turn prevented the excess in oxidative stress, as evidenced by the decrease in oxDJ1, 8OHdG, and mitochondrial accumulation (Fig. 6p–s).

Collectively, our results demonstrate that high and persistent PIAS2 expression is an important driver of the PD-like pathology that is observed in *Ifnb*^*–/–*^ mice. Conversely, siRNA against PIAS2 restores the activity of pERK1/2-pP53 and the anti-redox activity of DJ1, preventing oxidative damage and mitochondrial dysfunction. These findings also reinforce that in vivo high expression of PIAS2 induces cognitive impairments and its knockdown improves cognition in the *Ifnb*^*–/–*^ PDD model (Supplementary Fig. S4).

## Discussion

Our aim in the current study was to recognize signaling pathways that are disrupted in sPD and its progression to dementia in sPDD. By transcriptomic analysis, we first examined the gene expression profiles of sPD patients by GSEA. By combining four independent transcriptomic datasets from sPD patients to create a discovery cohort, we found that the cytokine–cytokine receptor and its related JAK-STAT pathways were the most highly dysregulated pathways compared with healthy individuals. Moreover, to narrow the pathway, we identified strong association of sPD with dysregulated type I IFN signaling among the cytokine–cytokine receptor pathway. Furthermore, these data cement the relevance of defective type I IFN signaling to sPD, especially sPDD, given our findings that the lack of *Ifnb* or *Ifnar1* in mice [9] leads to clinical features that resemble sPDD. Our additional analysis revealed that select genes in type 1 IFN signaling have a robust link to sPD and dementia. While some individual proteins involved in the type I IFN pathway had been studied in relation to PD, such as STING linked to mitochondrial dysfunction [56], it is the first time that dysfunctional type I IFN signaling pathway as whole is found associated with sPD.

Among the confirmed genes in the type I IFN signaling pathway, we identified PIAS2, the specific negative regulator of the IFNβ-IFNAR signaling [22], i.e., via specific inhibition of STAT2 [21] to impact the survival and function of neurons. Moreover, PIAS2 was significantly raised in neurons in the brains of sPD, and in particular in sPDD patients, paralleling our finding in *Ifnb*^*−/−*^ mice. By knocking down PIAS2 in the brain of *Ifnb*^*−/−*^ mice, the PD pathology and clinical manifestations were reversed. We next established that high expression of neuronal PIAS2 in vivo was sufficient to cause PDD-like pathology, and behavioral defects including cognitive impairments in healthy mice. PIAS2 overexpression triggered mitophagy block, resulting in damaged mitochondrial accumulation and elevated oxidative stress including increased oxidative DJ1, and in turn it inhibited ERK1/2 and P53 signaling by reducing their phosphorylation (Supplementary Fig. S4).

Our current findings are significant particularly because the molecular understanding of PD has been based primarily on the discovery of rare familial gene mutations, and although sporadic disease constitutes up to 95% of all PD cases [5, 6] and has been associated with such genes, its etiology remains unknown. Degenerative PD is generally regarded as a movement disorder, but up to 80% of patients will also develop dementia [57]. The importance of functional dopamine and dopamine-producing neurons in relation to movement disorder has received significant attention in the disease progression, but the underlying molecular mechanisms that drive the advancement from the loss of DA neurons to dementia are poorly understood.

Recent advances in genotyping by GWASs have enabled genetic risk factors that are associated with familial PD to be identified, but no evident gene or molecular factor has surfaced in the onset and progression of idiopathic disease. Furthermore, if overly stringent criteria are applied in GWASs, significant genes with an otherwise weak association with the pathology could be inadvertently disregarded, particularly in sporadic PD. Thus, if several such genes cooperate in a signaling pathway and collectively induce the PD phenotype, they would not be detected.

According to the IPDGC, when loci with weak association are included in the analysis of a GWAS, the estimated heritability of PD increases from approximately 4–27% [41]. This finding suggests that much of its genetic association lies below the thresholds for significance in the primary analysis, causing many true polygenic risk alleles to be overlooked [58]. Furthermore, PD is precipitated by a minor change in α-syn expression in patients with familial α-syn gene duplication [59] and perhaps by environmental factors that cause the complete dysfunction of otherwise subtle gene defects that coordinate in a given signaling pathway. These data indicate that sPD is not necessarily induced by genes with strong effects, underscoring the need to identify and consider weaker disease-associated genes as an entity. To this end, GSEA is a useful tool for discovering the relevant pathways and genes that predispose one and contribute to PD pathology.

In this study, we tested the hypothesis that sPD arises from the dysregulation of one or more genes, individually or cooperatively, in a specific signaling pathway which determines sPD and its progression to dementia in sPDD. Upon identifying dysregulated type I IFN signaling pathway in sPDD, to further characterize candidate disease-associated genes that had sequence variants in this pathway, we performed a meta-analysis of earlier GWASs on PD patients and detected many factors in IFNAR signaling that were enriched versus HCs. Thus, although these variants did not pass the stringent significance thresholds in the original GWASs, our results imply that this category of genes is pertinent to sPD.

Through transcriptomic analysis, we identified PIAS2 upregulation in all sPD patients with further increase in sPDD patients and established its pathogenic role. There are little data on the involvement of PIAS2 in PD pathology, and its contribution to PD and dementia has not been established. The JAK-STAT pathway, of which PIAS2 is a downstream effector [21], has been suggested to be associated with neurogenesis and to elicit neuroprotective signaling, but its blockade reduces microglia-mediated neuroinflammation in a model of PD [60, 61]. Furthermore, PIAS2 drives postsynaptic dendritic morphogenesis [62], albeit pertaining more to brain development and plasticity. These findings have created ambiguity with regard to the actual function of this pathway in PD, necessitating the identification of the molecular defects and pertinent cell types in this disease.

Consequently, we determined the contribution of PIAS2 to PDD. Neuronal expression of PIAS2 in the SN, STR, and prefrontal cortex of WT mice caused significant behavioral defects, including movement disorders, cognitive decline, and the loss of dopaminergic neurons—main hallmarks of PDD. Furthermore, we observed that PIAS2 has a negative impact on neuronal action potentials, which is compatible with its potential role in driving the cognitive decline in sPDD.

Based on these findings, our newly identified dysfunctional type 1 IFN signaling pathway, via PIAS2, is the first revelation of the involvement of an entire signaling cascade in the evolution of PD, including its development to dementia. Also, PIAS2 has been reported to interact functionally or physically with many components in this pathway, albeit in contexts other than PD. Based on our data, PIAS2 has potentially compartment-specific functions in neurons, regulating IFNβ-IFNAR signaling in the nucleus and cytoplasm with pERK1/2 and pP53 [63] as partners. For example, PIAS2 could negatively regulate type 1 IFN target genes by blocking STAT2 while governing the transcriptional expression of such genes as *PARK7* [64], *UCP2* [65], and *TH*, which are important for the regulation of neuronal oxidative phosphorylation and DA neuron survival. This differential regulation is consistent with the activity of PIAS2 in upregulating or downregulating target genes in MAPK signaling, depending on whether it is activated by ERK or p38 signaling [66]. A role for pERK/pP53 is also reported in autophagy regulation [52, 53]. In support, we observed that neuronal PIAS2OE upon blocking phosphorylation of ERK/P53 was associated with auto/mitophagy block and hence accumulation of damaged mitochondria. Previously, we have shown that neuronal lack of IFNβ-IFNAR signaling blocks auto/mitophagy and results in lack of cellular α-syn clearance, and hence its pathological accumulation [9, 32, 67, 68], In support, here we confirmed that PIAS2OE contributes to the accumulation of pathological α-syn and dysfunctional mitochondria with raise in oxidative stress markers, like oxDJ1, associated with inducing a mitophagy block, a process linked to neurodegenerative diseases including PD [69, 70].

PIAS2 is reported to regulate DJ1 activity [50], a transcriptional modulator, notably of P53 [71] and ERK [72], and antioxidant that has been linked extensively to PD. DJ1 has been reported independently to be a PD-associated protein, and the DJ1 L166P mutation is a rare genotype of the disease [73, 74]. The L166P mutation renders DJ1 improperly SUMOylated and prone to aggregation, causing it to colocalize with tau and α-syn in patients with Pick disease and multiple system atrophy, two neurodegenerative diseases [75, 76]. In our study, PIAS2OE in neurons upregulated *DJ1* mRNA while increasing primarily its oxidation; accordingly, oxDJ1 levels are elevated in PD as a result of oxidative stress [77]. DJ1 governed by PIAS2 might act as an antioxidant by activating signaling factors, such as p53, to restore the redox balance [71]. Independently of DJ1, ERK signaling has been suggested to control p53 activity and thus regulate DNA damage-induced neuronal death [78]. Although our data presented here strongly support a major role for PIAS2 by blocking the classical IFNβ-IFNAR signaling pathway, i.e., counteracting JAK-STAT2, pERK/pP53, its downstream mitophagy and upregulating oxidative stress including oxidation of DJ1, a role for PIAS2 through SUMOylation regulation could not be excluded, particularly given the pleiotropism of the type I IFN pathway.

Thus, our findings imply that the dysregulation of PIAS2 potentiates the development of PD on several levels against an array of signaling proteins, the multifaceted functions of which converge to disrupt neuronal homeostasis and functions. Collectively, our novel findings render PIAS2 an exciting target for future therapeutic approaches in PD.

## Supplementary information

supplementary figure legends

Supplementary Figure 1

Supplementary Figure 2

Supplementary Figure 3

Supplementary Figure 4

Data file 1

Data file 2

Data file 3
