# Supplementary material for: PIAS2-mediated blockade of IFN-β signaling: a basis for sporadic Parkinson disease dementia
Source: Mol Psychiatry. 2021 Jul 8;26(10):6083–99. doi: 10.1038/s41380-021-01207-w (PMC8758491; doi:10.1038/s41380-021-01207-w)
Supplement: Supplementary file 1 — supplementary figure legends [file 41380_2021_1207_MOESM1_ESM.docx]

**Supplementary Figure legends**

**Figure S1. *Ifnb­^–/–^* neuronal gene signature distinguishes sPD with dementia from non-dementia and healthy controls. a-b)** Enrichment plots distinguishing sPDD from HC and sPDND based on core enriched gene signature in the Cytokine-cytokine receptor interaction pathway when comparing **a)** *Ifnb^–/–^* vs. *Ifnb^+/+^* neurons and **b)** *Ifnb^–/–^* vs. rIFN-β–treated (100U/ml for 24 hours) *Ifnb^–/–^* neurons (GSE63815). NES: normalized enrichment score. **c)** Interactive signaling map showing regulated genes in the type I IFN signature comparing PD and HC from pooled cohorts GSE7621, GSE20141, and GSE49036. **d)** IF staining for PIAS2 (green), βIII-tubulin (red), and DAPI in prefrontal cortex of WT, *Ifnb^–/–^*, *Ifnar1^–/–^*, and *nes^Cre^:Ifnar1^fl/fl^* mice. Bar, 50 μm.

**Figure S2.** **PIAS2 overexpression is sufficient to cause neurodegeneration, motor and cognitive impairments, and PD-like dementia in mice. a-b)** Western blot of mCherry in mice injected with AAV6 PIAS2-mCherry or AAV6 mCherry CTR in the nigrostriatal region and cortex. Vinculin was used as a loading control, and data are normalized to mCherry control±SEM; ∗∗*p* < 0.001 by unpaired student’s t-test, n=4. **c)** IF of Nissl (yellow) and PIAS2 (red) in brainstem of WT mice injected with AAV6 PIAS2-mCherry or AAV6 mCherry control. Scale bar, 14 μm. **d)** WT, *Ifnb^–/–^*, and *Ifnar1^–/–^* mice injected with AAV6 PIAS2-mCherry or AAV6 mCherry CTR. Cognition tested in Barnes maze on Day 30 postinjection. Data are represented in seconds as time spent in the entry zone ±SEM. ∗*p* < 0.05, ∗∗*p* < 0.01 by paired student’s t-test in the same group among 1 and 2-3 trails. n=6 /WT-CTR, 7 /WT-PIAS2, n=4/ in each mCherry CTR- and PIAS2-mCherry-injected group of *Ifnb^–/–^* and *Ifnar1^–/–^* mice; total n=29. **e)** RT-qPCR quantification of TH in the brainstem normalized to *GAPDH* ±SEM in AAV6 PIAS2-Cherry or AAV6 mCherry control groups; ∗*p* < 0.05 by unpaired student’s t-test. n=3. **f-l)** AAV6 hSNCA/AAV6 PIAS2-mCherry or AAV6 hSNCA/mutPIAS2-mCherry or CTR-injected WT mouse brains. **f)** Latency to the first rearing event by cylinder test on Day 16 postinjection shows anxiety. ∗∗*p* < 0.001 by unpaired student’s t-test. n=6/group. **g-h)** Western blot of PIAS2-mCherry and hSCNA in brainstem of mice. Vinculin was used as a loading control. **h)** Quantification of **g)** normalized to mCherry control±SEM; ∗∗*p* < 0.01 by unpaired student’s t test. n=3-5. **i-j)** Western blot of pα-syn and total α-syn in brainstem of mice. Vinculin was used as a loading control. **i)** Quantification of **j)** normalized to mCherry control±SEM; ∗*p* < 0.05, ∗∗*p* < 0.01 by unpaired student’s t test. n=3. **k)** IF staining for pα-syn (red), Hsp60 (green), Nissl (yellow), and DAPI in brainstem **l)** RT-qPCR for *DJ1*, *SOD1*, and *UCP2* using WT mouse brainstem overexpressing PIAS2-mCherry or mCherry CTR normalized to *GAPDH*±SEM; **p* < 0.05, ***p* < 0.01 by student’s t-test. n=3. **m)** IF staining for Hsp60 (green) and DAPI (blue) in CN. **n)** IF staining for TOM20 (red) in brainstem. **o)** LC3B blot and quantification from brainstem of mice injected with AAV6 PIAS2-mCherry or AAV6 mCherry CTR. **p)** LC3B blot and quantification from AAV6 hSNCA/AAV6 PIAS2-mCherry or AAV6 hSNCA/mutPIAS2-mCherry or CTR-injected WT mouse brainstems. **q)** Blot anti SUMO1 and quantification from AAV6 hSNCA/AAV6 PIAS2-mCherry or AAV6 hSNCA/mutPIAS2-mCherry or CTR-injected WT mouse brainstems. For p and q, CTR and mutPIAS2 have been pooled as no differences could be observed between these 2 controls.

**Fig. S3. siPIAS2 decreases accumulation of damaged mitochondria in the *Ifnb^−/−^* PD mouse model. a)** Blot anti SUMO1 and quantification from siPIAS2- vs. siCtrl-treated brainstems. **b)** IF staining for 8OHdG (green) in striatum and prefrontal cortex of *Ifnb^–/–^* mouse brain after knockdown of PIAS2 or scrambled control. **c)** IF staining of oxidized DJ1 (green), TOM20 (red), Nissl (yellow), and DAPI (blue) in prefrontal cortex of *Ifnb^–/–^* mice after knockdown of PIAS2 or scrambled control. Graphs represent mean fluorescence in oxDJ1^+^Nissl^+^ and TOM20^+^Nissl^+^ neurons, normalized to scrambled control; **p* < 0.05, ***p* < 0.01 by student’s t-test. n=9-10/group for oxDJ1 and n=7-8/group for TOM20. **i-s)** *In vitro* culture of *Ifnb^–/–^* primary cortical neurons (CNs) on siRNA knockdown of PIAS2 (siPIAS2) or scrambled control (siCtrl).

**Fig S4. Defects in type I IFN signaling via elevated PIAS2 found in sPD with dementia induces neurodegeneration.**

Type I IFN signaling pathway as a whole is associated with sPDND and sPDD. Specifically, patients present alterations of IFNAR, TYK2, STAT1/2 and overactivation of PIAS2, an inhibitor of type I IFN signaling pathway. These defects cause neuronal pathology including neurite retraction, disturbed neuronal excitability and neurodegeneration. In particular, type I IFN signaling pathway activates autophagy and mitophagy. Upon pathological conditions, its inactivation inhibited ERK and p53 phosphorylation, induced autophagy/mitophagy block, increased mitochondrial mass accompanied with elevated ROS production and oxidated DJ1. This further led to accumulation of pα-synuclein. Such molecular dysfunctions were mimicked in mice by overexpression of PIAS2 that led to development of PDD-like pathologies and cognitive and motor impairments. In the contrary, the knockdown of PIAS2 in *Ifnb*^−/−^ PDD-model reversed these PDD-like pathologies and clinical manifestations.
