# Supplementary figures and images for: PIAS2-mediated blockade of IFN-β signaling: a basis for sporadic Parkinson disease dementia
Source: Mol Psychiatry. 2021 Jul 8;26(10):6083–99. doi: 10.1038/s41380-021-01207-w (PMC8758491; doi:10.1038/s41380-021-01207-w)

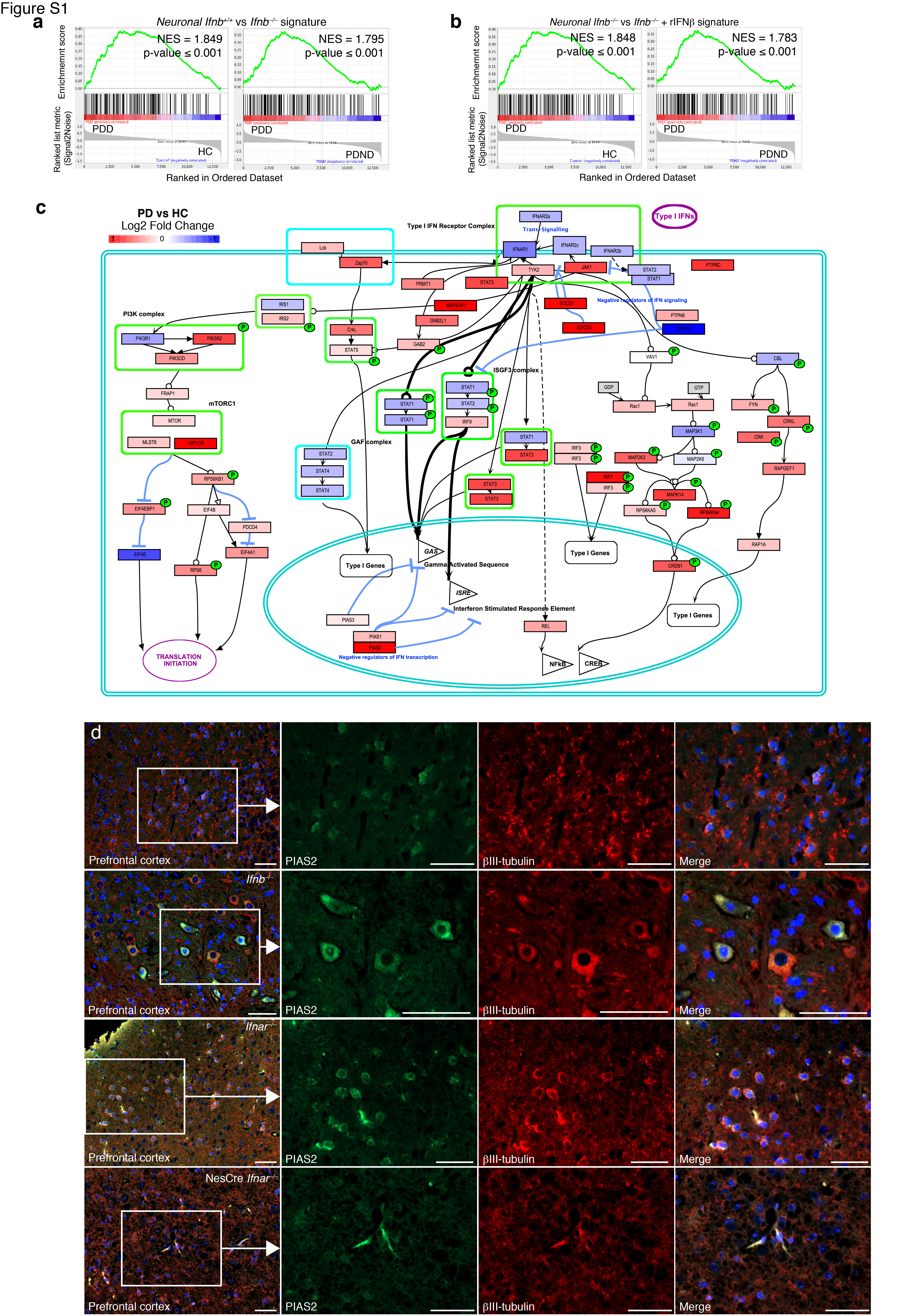

Supplement: Supplementary file 2 — Supplementary Figure 1 [file 41380_2021_1207_MOESM2_ESM.jpg]

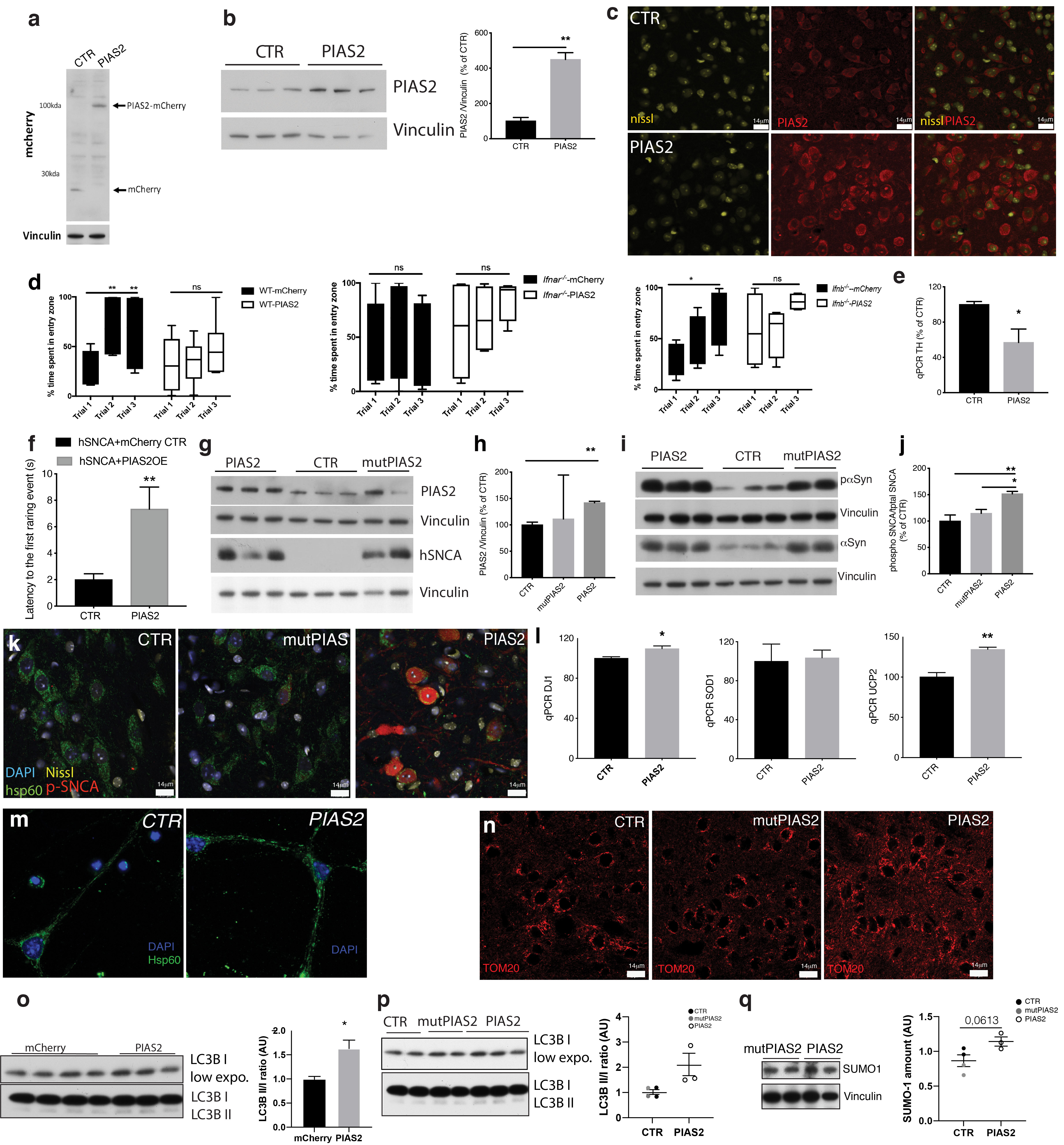

Supplement: Supplementary file 3 — Supplementary Figure 2 [file 41380_2021_1207_MOESM3_ESM.jpg]

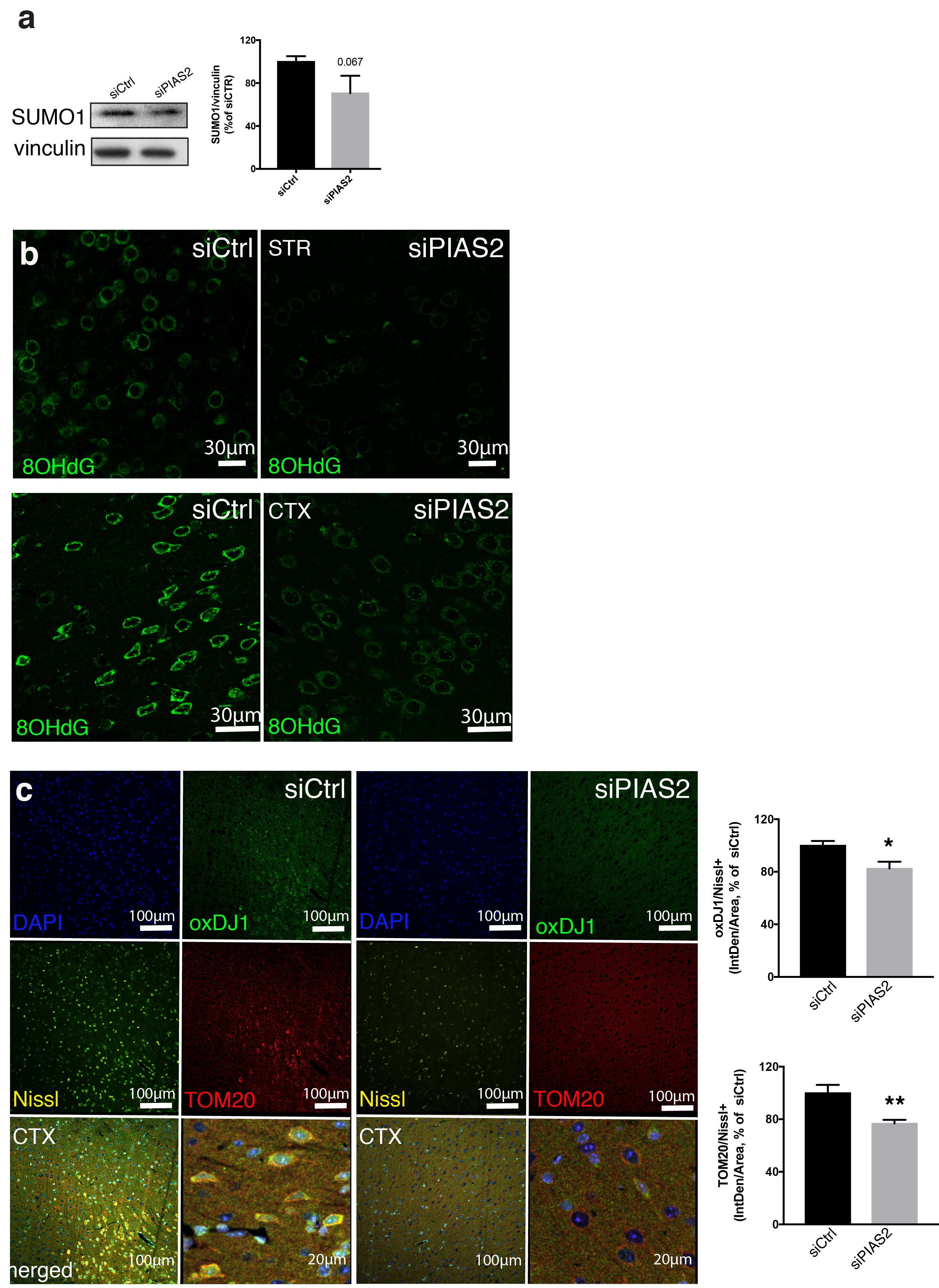

Supplement: Supplementary file 4 — Supplementary Figure 3 [file 41380_2021_1207_MOESM4_ESM.jpg]

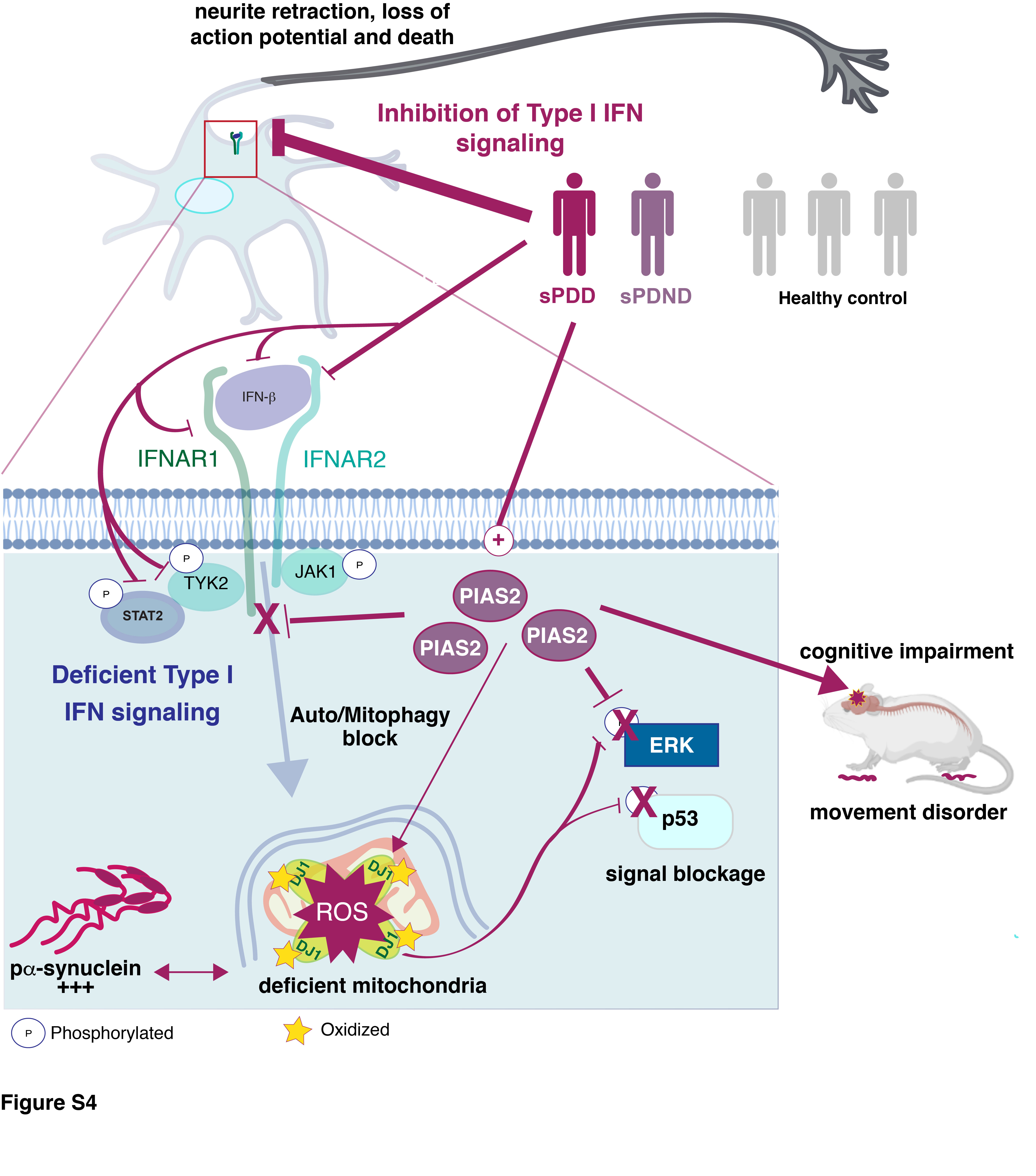

Supplement: Supplementary file 5 — Supplementary Figure 4 [file 41380_2021_1207_MOESM5_ESM.jpg]
